# Identification and Characterization of Extracellular Vesicles and Its DNA Cargo Secreted During Murine Embryo Development

**DOI:** 10.3390/genes11020203

**Published:** 2020-02-17

**Authors:** Blanca Simon, David Bolumar, Alicia Amadoz, Jorge Jimenez-Almazán, Diana Valbuena, Felipe Vilella, Inmaculada Moreno

**Affiliations:** 1 Igenomix Foundation-INCLIVA Biomedical Research Institute, Ronda de Narcís Monturiol, 11B, 46980 Paterna, Spain; blansi@alumni.uv.es (B.S.); david.bolumar@igenomix.com (D.B.); 2 Department of Pediatrics, Obstetrics and Gynecology, School of Medicine, University of Valencia, Av. de Blasco Ibáñez, 15, 46010 Valencia, Spain; 3 Igenomix, R&D, Parque Tecnológico de Paterna, Ronda de Narcís Monturiol, 11B, 46980 Paterna, Spain; alicia.amadoz@igenomix.com (A.A.); jorge.jimenez@igenomix.com (J.J.-A.); diana.valbuena@igenomix.com (D.V.)

**Keywords:** extracellular vesicles, exosomes, microvesicles, apoptotic bodies, DNA, preimplantation embryos, murine blastocysts

## Abstract

Extracellular vesicles (EVs) are known to transport DNA, but their implications in embryonic implantation are unknown. The aim of this study was to investigate EVs production and secretion by preimplantation embryos and assess their DNA cargo. Murine oocytes and embryos were obtained from six- to eight-week-old females, cultured until E4.5 and analyzed using transmission electron microscopy to examine EVs production. EVs were isolated from E4.5-day conditioned media and quantified by nanoparticle tracking analysis, characterized by immunogold, and their DNA cargo sequenced. Multivesicular bodies were observed in murine oocytes and preimplantation embryos together with the secretion of EVs to the blastocoel cavity and blastocyst spent medium. Embryo-derived EVs showed variable electron-densities and sizes (20–500 nm) and total concentrations of 1.74 × 10^7^ ± 2.60 × 10^6^ particles/mL. Embryo secreted EVs were positive for CD63 and ARF6. DNA cargo sequencing demonstrated no differences in DNA between apoptotic bodies or smaller EVs, although they showed significant gene enrichment compared to control medium. The analysis of sequences uniquely mapping the murine genome revealed that DNA contained in EVs showed higher representation of embryo genome than vesicle-free DNA. Murine blastocysts secrete EVs containing genome-wide sequences of DNA to the medium, reinforcing the relevance of studying these vesicles and their cargo in the preimplantation moment, where secreted DNA may help the assessment of the embryo previous to implantation.

## 1. Introduction

Embryo-endometrial communication is mediated by different mechanisms including extracellular vesicles (EVs) with a plethora of molecules released to this interface. EVs constitute a novel bidirectional form of cross-talk during embryo implantation, as they are secreted by both the endometrium [1,2], and the embryo [3,4] contributing to new functional perspectives as their cargo is composed by virtually all sorts of biomolecules. In fact, EVs have been described to participate in several reproductive processes, such as gametogenesis, fertilization, embryo development and implantation, and fetal development throughout term (for review see [5]).

EVs are secreted by cells of different human tissues and organs and can be isolated in a variety of biological fluids including blood [6], urine [7], saliva [8], breast milk [9], amniotic fluid [10], ascitic fluid [11], cerebrospinal fluid [12], bile [13], semen [14], and endometrial fluid [2].

Three major types of EVs have been described based on their biogenetic pathway, composition, and physical characteristics such as size or density, namely, apoptotic bodies (ABs), microvesicles (MVs), and exosomes (EXOs) [15,16]. ABs result from the outward budding of the plasma membrane in the context of programmed cell death and their diameter ranges from 50–5000 nm [17,18]. MVs are produced directly by outward budding of the plasma membrane. They are in the size range from 100–1000 nm and are associated to the GTP-binding protein ARF6, which is used for their identification [19]. EXOs originate from the endosomal pathway as late endosomes that evolve into multivesicular bodies and migrate from the perinuclear region to the cell surface by fusion [20,21]. Their sizes range between 30–150 nm and are identified by classical molecular markers such as tetraspanins (CD63, CD9, CD81), flotillin-1, or heat shock 70-kDa proteins, although recent reports considered that other EVs might share these markers [22]. Their cargo portrays their functionality in regulating communication by means of proteins, cytokines, lipids, RNA, or DNA [23].

The embryo also secretes EVs that participate in both the dialogue with the maternal endometrium [24], and in self-paracrine regulation [4]. EVs are secreted by a trophectoderm cell line in the pig model [25], and in the human model [26] stimulating the proliferation of endothelial cells in vitro, thus becoming potential regulators of maternal endometrial angiogenesis. Regarding the nucleic acid cargo of embryo derived EVs, several papers have described the RNA cargo of trophoblast-derived EVs, mainly focusing on miRNA [25], linking miRNA signature with successful pregnancy [27], nevertheless, the DNA cargo of embryo derived EVs has received little attention. The aim of the present study is to identify and characterize embryo derived EVs secreted during murine embryo development, and to analyze their DNA cargo comparatively among the different EVs subpopulations.

## 2. Materials and Methods

### 2.1. Mouse Embryo Isolation and Culture 

Female B6C3H1/Crl mice of 8-weeks of age (Charles River Laboratories International, Wilmington, MA, USA) were stimulated using 10 IU of Foligon/PMSG (MSD Animal Health, Madrid, Spain) followed by administration of 10 IU of Ovitrelle/hCG (Merck Serono, Darmstadt, Germany) 48 h later. Female mice were mated with males overnight and sacrificed by cervical dislocation 24 h after vaginal plug was observed. At embryonic day 1.5 (E1.5), embryos were extracted by flushing the oviduct with PBS using a 30G syringe needle. Non-fertilized metaphase II (MII) oocytes were also collected. Mouse embryos were washed and cultured in oxygenated G2-Plus media (Vitrolife, Västra Frölunda, Sweden) at 37 °C and 5% CO2 in groups of 40 embryos per well (Nunc 4-well plates, ThermoFisher Scientific, Whaltham, MA, USA) until day E4.5, reaching the stage of hatching/hatched blastocysts. MII oocytes, embryos at different developmental stages (E2.5, E3.5, and E4.5) and spent culture media from them were collected and preserved accordingly to the objective of the investigation.

The total number of females used was 80. Embryos from 10 animals were used for identification and phenotypic characterization of EVs using electron microscopy and immunogold. Ten animals were used in triplicates (n = 30) for quantification by nanoparticle tracking analysis and 10 in quadruplicates (n = 40) for sequencing experiments. An average of 32 embryos were obtained per female, 50% of them achieved hatching/hatched blastocyst stage by day E4.5. All animal experimentation was conducted at the animal facility located in the School of Pharmacy at the University of Valencia under the protocol code 2015/VSC/PEA/00048, approved by the Ethics Committee of Animal Welfare on 12 March 2015.

### 2.2. Isolation and Purification of EVs Secreted by Murine Embryos 

Conditioned media from embryo culture was collected at day E4.5 and centrifuged at low speed (300× *g*, 10 min) to remove larger debris. The resulting supernatant was centrifuged at 2,000× *g* for 10 min to recover ABs as previously described [28]. It was subsequently ultracentrifuged at 185,000× *g* for 70 min in a P50A3 Hitachi rotor (Hitachi, Tokio, Japan) to collect non-apoptotic EVs (naEVs) that includes MVs and EXOs in the same fraction. All centrifugations were conducted at 4 °C.

### 2.3. Transmission Electron Microscopy and Immunogold

Oocytes and embryos at different developmental stages (E2.5, E3.5, and E4.5) and pellets containing EVs from spent culture media were fixed overnight in Karnovsky’s solution (2.5% glutaraldehyde/2% formaldehyde in phosphate buffer 0.1 M, pH 7.4), washed in PBS and encapsulated in 2% agar in distilled water. Samples were then washed 5 times in PBS for 5 min and stained in a 2% osmium tetroxide 0.2 M PBS solution for 2 h. Then, dehydrated following the next sequence: three washes of 5 min in distilled water at 4 °C, 5-min wash in 30° ethanol, 10-min wash in 50° ethanol, 10-min wash in 70° ethanol twice, 45-min wash in 2:1 90° ethanol + LR-white twice, 45-min wash in 2:1 100° ethanol + LR-White, and O/N wash in 1:2 100° ethanol + LR-white in continuous shaking. Ethanol was let to evaporate, and wash media was changed by 100% LR-White in continuous shaking, for a 30-min incubation. Finally, samples were allowed to polymerize for 1 day at 60 °C, protecting them from the air. Resin-embedded samples were ultrasectioned in 60 nm slices, incubated for 1 h on formvar carbon-coated cooper (regular visualization) and nickel (immunogold visualization) grids, and contrasted with uranyl acetate. Ultrathin cuts were done using a UC6 Leica ultramicrotome (Leica, Wetzlar, Germany) equipped with an Ultra 45° diamond blade (Diatome, Hatfield, PA, USA). Prepared samples were observed using a JEM-1010 transmission electron microscope (Jeol Korea Ltd., Seoul, South Korea) at 80 kV, using a digital camera MegaView III and Olympus Image Analysis Software.

For the immunogold labelling assays, 60 nm sections from previous resin blocks were processed as described by Marcilla and collaborators [29]. Specifically, two different combinations of primary antibodies were used: rabbit α-CD63 (Abcam, ref: ab118307), rabbit α-ARF6 (Abcam, ref: ab77581), and mouse α-DNA (Millipore, Burlington, MA, USA ref: CBL186). All the antibodies were diluted in PBS/0.5% BSA following the manufacturer’s datasheets. Then, the grids were washed in PBS/0.5% BSA and incubated with gold-coupled secondary antibodies [goat α-mouse coupled to 10 nm gold particles (BBI solutions, Crumlin, UK, ref: 15736) and goat α-rabbit coupled to 18 nm gold particles (Jackson Immunoresearch, Ely, UK) at 1:20 dilution in PBS/0.5% BSA for 30 min, following datasheets specifications. In parallel, paired grids were incubated only with the secondary antibodies as negative controls. Finally, grids were stained with 2% uranyl acetate and imaged by TEM as previously described.

### 2.4. Nanoparticle Tracking Analysis 

Nanoparticle tracking analysis (NTA) was performed using a NanoSight300 (NS300, Malvern Instruments ltd, Malvern, UK). The naEVs fraction isolated from E4.5 spent media was resuspended in 1 mL of sterile PBS w/o Ca2+/Mg2+ (Biowest, Nuaillé, France, ref. L0615-500). In parallel, an aliquot of fresh embryo culture media was processed as a blank control. Three 60 sec videos were recorded under the static flow conditions for each sample with camera level set at 11. Videos were analyzed with NTA software version 3.2 Dev Build 3.2.16 to determine mean size and estimated concentration of measured particles with corresponding standard error. The NS300 system was calibrated with silica microspheres 100, 167, and 300 nm (Bangs Laboratories, Inc.; Fishers, IN, USA) prior to analysis, as previously demonstrated [30], auto settings were used for blur, minimum track length, and minimum expected particle size.

### 2.5. DNA Amplification and Sequencing of EVs Derived from Murine Embryos

Sequencing analysis was conducted to assess whether a specific DNA cargo is loaded into EVs secreted by the embryo, namely, ABs and naEVs. Mouse embryos representing the whole murine genome and EVs-depleted supernatant fraction after isolation of EVs (SN) were also sequenced. Because ABs are the result of dying cells, it is expected to have a representation of the total embryo DNA in this fraction. Sequencing of the DNA of ABs was used as an internal control. The negative control was blank medium that had not been in contact with murine embryos (blank). Groups of 10 mice were used to obtain the samples (embryo, ABs, naEVs, SN) in four independent sequencing experiments. DNase treatment of the different EV populations was conducted to evaluate and remove external DNA contamination and its potential bias in the analysis. The samples corresponding to DNase treated and untreated ABs from one of the four replicates were lost due to technical reasons, so only DNA from embryos, treated and untreated naEVs, and SN were included in the analysis for this biological replicate.

Embryos for DNA sequencing corresponding to E4.5 stage, were snap-frozen for initial DNA extraction and kept at −80 °C until processing. At this point, embryos were diluted in nuclease-free water (Ambion, ThermoFisher Scientific, Waltham , MA, USA) at a rate of 1 embryo/µL. DNA from pooled SN from all embryo culture wells and an equal volume of fresh blank media (G2-Plus) was extracted using QIAamp DNA mini kit (Qiagen, Hilden, Germany), eluting in 30 µL of nuclease free-water. For DNase treatment, ABs and naEVs were separated in two equivalent aliquots from pooled spent embryo culture media at the beginning of the isolation process. Once isolated, EVs were resuspended in nuclease-free water and, in the case of DNase treatment, 50 U/mL DNaseI (Sigma-Aldrich, San Luis, MO, USA) in 20 mM Tris-HCl (Thermo Fisher Scientific), 10 mM MgCl2 (Thermo Fisher Scientific), and 1 mM CaCl2 (Sigma-Aldrich, ref. 21115-100ML) were added to a final volume of 5 µL. Samples were then incubated at 37 °C for 30 min for DNase digestion followed by 10 min at 75 °C for DNase inactivation.

Immediately after, DNA amplification was performed using DOPlify whole genome amplification platform (RHS Ltd., Thebarton, Australia) following the manufacturer’s instruction. Then, DNA was purified by using AMPure XP (Beckman Coulter, Brea, CA, USA) at a final concentration of 1.8X, in order to recover small-sized DNA fragments, and eluted in a final volume of 20 µL of nuclease-free water. Subsequently, the amplified DNA profiles were analysed by TapeStation 4200 (Agilent, Santa Clara, CA, USA). Finally, samples were sequentially diluted to 0.5 and 0.2 ng/µL to meet libraries kit DNA input requirements by Qubit dsDNA HS Assay (TermoFisher Scientific, Waltham, MA, USA). DNA libraries were built using Nextera XT DNA Library Prep Kit, specifically designed for samples with low DNA input. The experimental procedure was conducted following the protocol provided by the manufacturer, adjusting AMPure XP purification to 1.8X proportion. Then, libraries were pooled and sequenced using a 300 cycles-NextSeq 500/550 v2 High Output cartridge in a NextSeq 550 platform (Illumina, San Diego, CA, USA). To do so, 5 μL of the libraries pool, normalized by the bead method, were diluted in 995 μL of HT1 buffer. Then, 750 μL of the dilution was rediluted in 750 μL of HT1 buffer. The resulting dilution was denaturalized at 98 °C for 4 min, cooled in ice for 5 min and loaded into the sequencing cartridge for sequencing.

### 2.6. Computational Analysis of Sequencing Results

Raw data pre-processing: Raw data from pair-ended Illumina sequencing was downloaded from Illumina BaseSpace and converted into FASTQ files using bcl2fastq (version 2.16.0.10). Then, each sample was aligned to the mouse reference genome (mm10) using BWA (version 0.7.10) [31]. Reads with mapping quality lower than 10 were filtered out using Samtools (version 1.1) [32] and duplicates were removed with PICARD software (version 1.119). Finally, the coverage of each genome feature was obtained with Bedtools (version 2.17.0) [33] using Ensembl Biomart mm10 annotations. The raw genomic sequences generated in this study are deposited in the SRA database under the accession number PRJNA547453.

Murine-specific DNA sequences: A greater than expected quantity of valid reads were observed in blank media. Using the blank media for a background subtraction with the count per million (CPM) of each gene feature to decrease the background noise is not usually recommended because this data transformation may break the statistical assumptions of later steps. Therefore, the approach used here was to identify and filter murine-specific reads in a common manner for all samples. Then, background noise is considered as a background population (blank) and included in the analysis as another comparison group, whose results could be considered as differences with the background. To identify murine-specific reads, pre-processed data was mapped to the human reference genome (Hg19) using BWA. Then, unmapped reads were filtered using Samtools [32] and read ids were retrieved. Murine-specific reads were retrieved from pre-processed data by read id using PICARD software. Finally, the coverage of each genomic feature was obtained with Bedtools using Ensembl Biomart mm10 annotations.

Differential DNA enrichment of vesicles’ cargo: The approach used for the differential DNA enrichment analysis is based on the edgeR methodology [34,35,36] that uses read counts of genomic features obtained from massively parallel sequencing technologies such as Illumina, 454, or ABI SOLiD applied to different types of experiments such as RNA-seq or ChIP-seq. edgeR can be applied to differential abundance analysis at the gene, exon, transcript, or tag level. In fact, read counts can be summarized by any genomic feature.

Here, differential DNA enriched regions were obtained using the following approach. In order to filter out lowly enriched regions, genomic features with greater than 1 cpm reads mapped in at least 1.5 samples (half of the mean of groups sizes) were kept for the following analyses. Raw counts were normalized using TMM method from edgeR R package [34]. A differential enrichment analysis of genome features was done using a generalized linear model approach for pairwise comparisons between sample types (embryo, ABs, naEVs, SN, blank). Experimental set and treatment were taken as factors in the additive model in order to adjust for differences between groups.

## 3. Results

### 3.1. Identification of EVs in Murine Oocytes and Embryos

**Figure 1:**
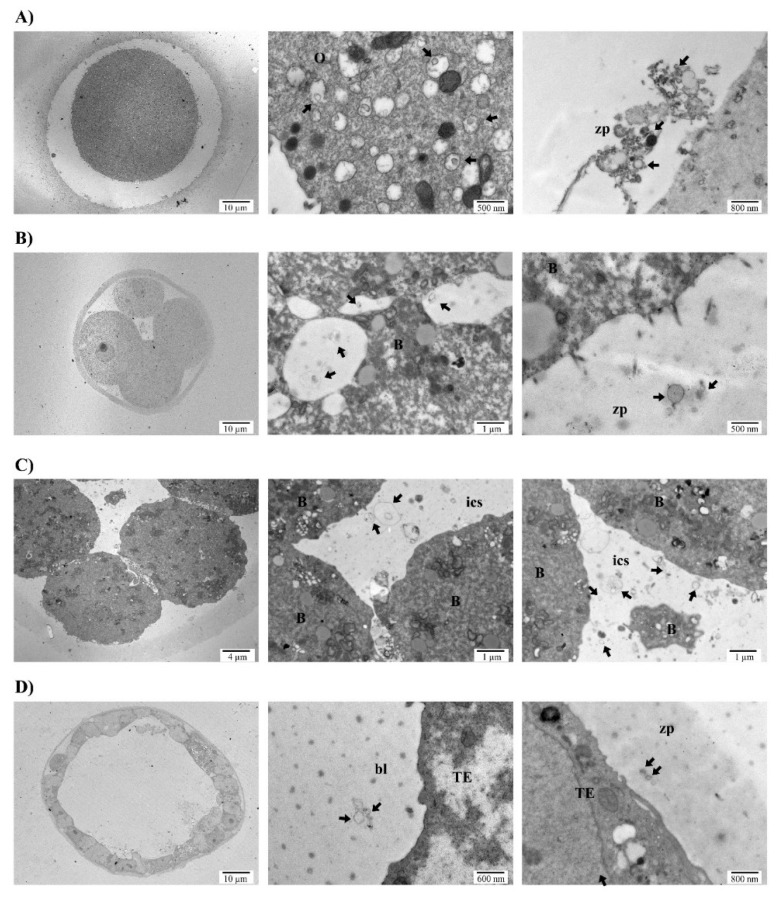
Vesicle production and secretion by murine embryos at different developmental stages shown by TEM. (**A**) Mouse oocyte (left) with magnifications of the production of multivesicular bodies (MVBs) in the cellular cytoplasm (center) and secretion of their content towards the zona pellucida (right). (**B**) E2.5 embryo (left) with magnifications of the cytoplasm containing MVBs (center) and the secretion of extracellular vesicles (EVs) through the zona pellucida (right). (**C**) E3.5 mouse embryo (left) showing MVB secreted to the intercellular space. (**D**) E4.5 blastocyst (left) secreting EVs into the zona pellucida (center) and blastocoel cavity (right). Abbreviations: B, blastomere; bl, blastocoel; O, oocyte; TE, trophectoderm cell; zp, zona pellucida.

Ultrastructural visualization of murine oocytes using TEM identified the existence of multivesicular bodies (MVBs) in the cytoplasm. These MVBs showed bilipid membrane vesicles containing smaller vesicular structures of different electron densities and sizes that would give rise to EXOs upon fusion of the MVBs with the oocyte plasma membrane. These structures were identified by their lower electron density, and vesicles of different sizes were observed entering the zona pellucida (Figure 1A). The presence of MVBs was also observed in the blastomeres at different embryo developmental stages (E2.5 and E3.5), migrating from the cytoplasm to the plasma membrane where their content was secreted outwards through the zona pellucida (Figure 1B). Interestingly, large vesicles including complex structures were also observed in the intercellular space (Figure 1C). At the blastocyst stage (day E4.5), the secretion of vesicular structures was observed both into the extracellular medium through the zona pellucida (zp), as well as into the blastocoel (bl) cavity (Figure 1D).

### 3.2. EVs Isolated from Blastocyst Culture Media

**Figure 2:**
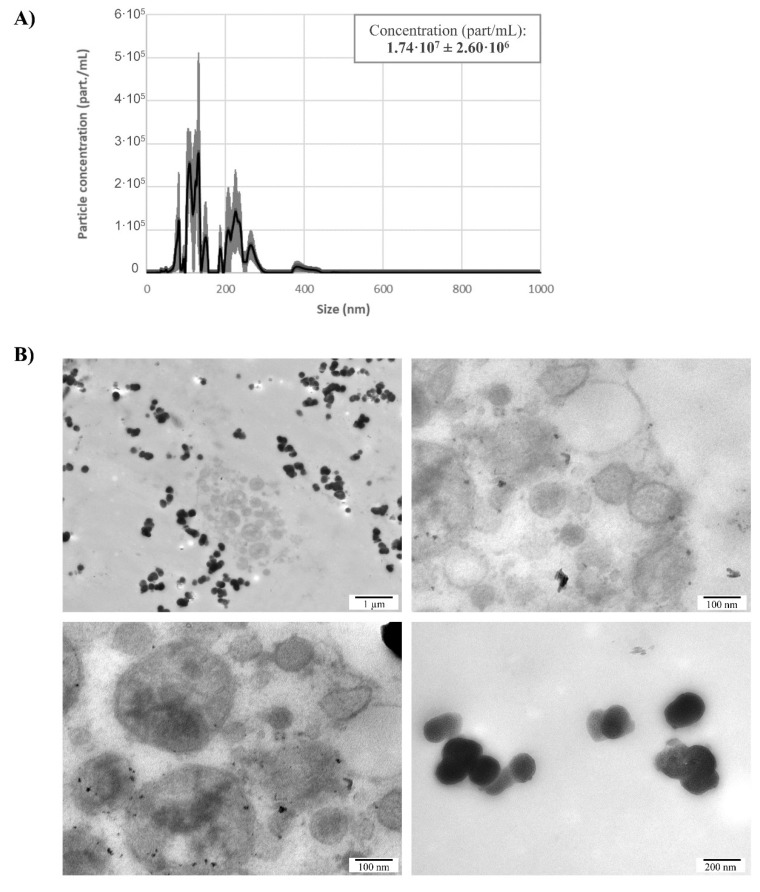
Isolation of naEVs from E4.5 blastocyst spent media. (**A**) Size distribution and quantification using nanoparticle tracking analysis of naEVs isolated from spent blastocyst media. Net particle concentration was calculated after subtracting particles found in the same volume of blank media. Values represent the mean of three independent experiments ±SEM. (**B**) Different images showing morphological characterization of naEVs in spent culture media by TEM. Particles of different aspects, electron densities and sizes ranging from 20–500 nm were observed.

In order to study naEVs-including MVs and EXOs-produced and released by murine blastocysts, spent media at developmental day E4.5 was ultracentrifuged and gathered naEVs where quantified by NTA. The vesicular fraction showed vesicle profiles compatible in size with that of EXOs and MVs. The total concentration of naEVs from spent media was 1.74·10^7^ ± 2.60·10^6^ particles/mL, with a size profile showing two main populations. A first population extended from approximately 50–170 nm and accounted for 9.87 × 10^6^ ± 7.55 × 10^5^ particles/mL (56.72%) while the second extended from 180–310 nm and were represented by 6.75 × 10^6^ ± 1.57 × 10^6^ particles/mL (38.83%) to the total (Figure 2A). Importantly, standard error of measurements showed that only a small amount of these particles remained constant among replicates (Figure 2A).

Finally, morphological analysis of EVs isolated from spent culture media by TEM showed vesicles of variable appearance, electron densities, and sizes ranging from 20 nm–500 nm, suggesting that EVs secreted by the embryo constitute a heterogenous population including different types of EVs (Figure 2B).

### 3.3. Phenotypic Characterization of Embryo Secreted EVs

**Figure 3:**
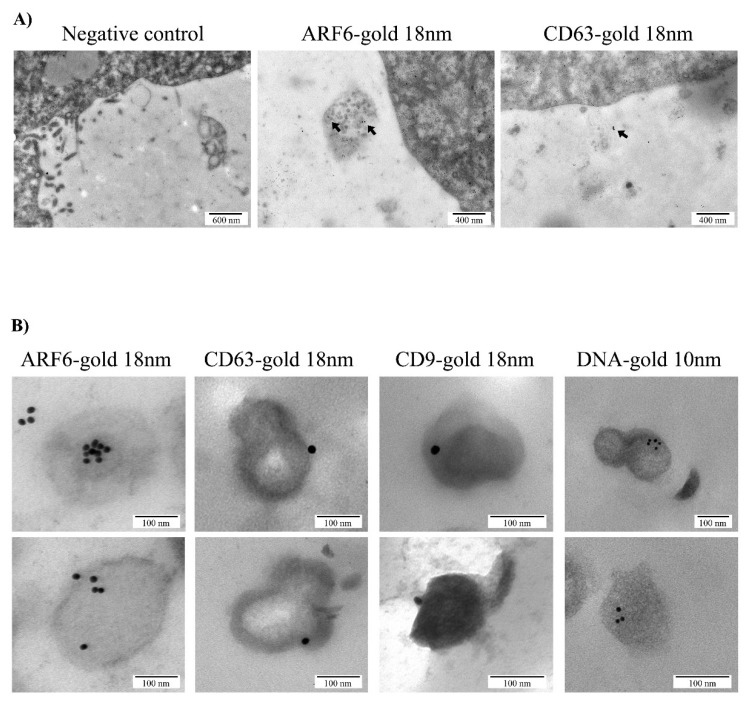
Phenotypic characterization of EVs using immunogold staining. (**A**) Vesicles secreted by the trophoectodermal (TE) cells of murine embryo blastocyst showed positive staining for microvesicles (MVs) and exosomes (EXOs) markers, ARF6 and CD63, respectively. (**B**) Immunogold detection of ARF6, CD63 and DNA in naEVs isolated from spent media of E4.5 blastocysts. ARF6 and CD63 are coupled to 18 nm gold particles; DNA is coupled to 10 nm gold particles. Bars represent 100 µm. Two representative images for each immunogold labeling experiment are included in the figure.

Phenotypic characterization of the EVs secreted by murine embryos to the spent medium was performed using immunohistochemistry coupled to gold nanoparticles using specific surface markers for EXOs (CD63) or MVs (ARF6). As a negative control, paired grids of E4.5 blastocyst were incubated with the secondary antibodies only. Positive ARF6 staining was consistent with an active vesicular biogenesis through phospholipase D pathway in embryos, a mechanism described for inward budding of MVBs membrane in EXOs biogenetic pathway [37] and outward budding of cell plasma membrane in MVs formation [38]. Positivity for CD63, a tetraspanin involved in the formation of EXOs, was considered as canonical marker for these types of EVs that originate from the endosomal pathway (Figure 3A).

Additionally, immunogold was performed on ultrathin sections to assess the presence of ARF6 and CD63 in naEVs fraction isolated by ultracentrifugation from E4.5 blastocyst spent media. In parallel, to study the potential cargo of DNA in naEVs, DNA targeting antibodies were used. The results revealed positivity for the microvesicular marker ARF6, exosomal marker CD63, and DNA (Figure 3B), thus suggesting the presence of DNA cargo in the EVs secreted by the embryos. Particularly, in our experimental set in which 702 single naEVs were studied, 16.1% of DNA positive EVs were identified.

### 3.4. Characterization of the DNA Cargo of the EVs Secreted by the Embryo

**Figure 4:**
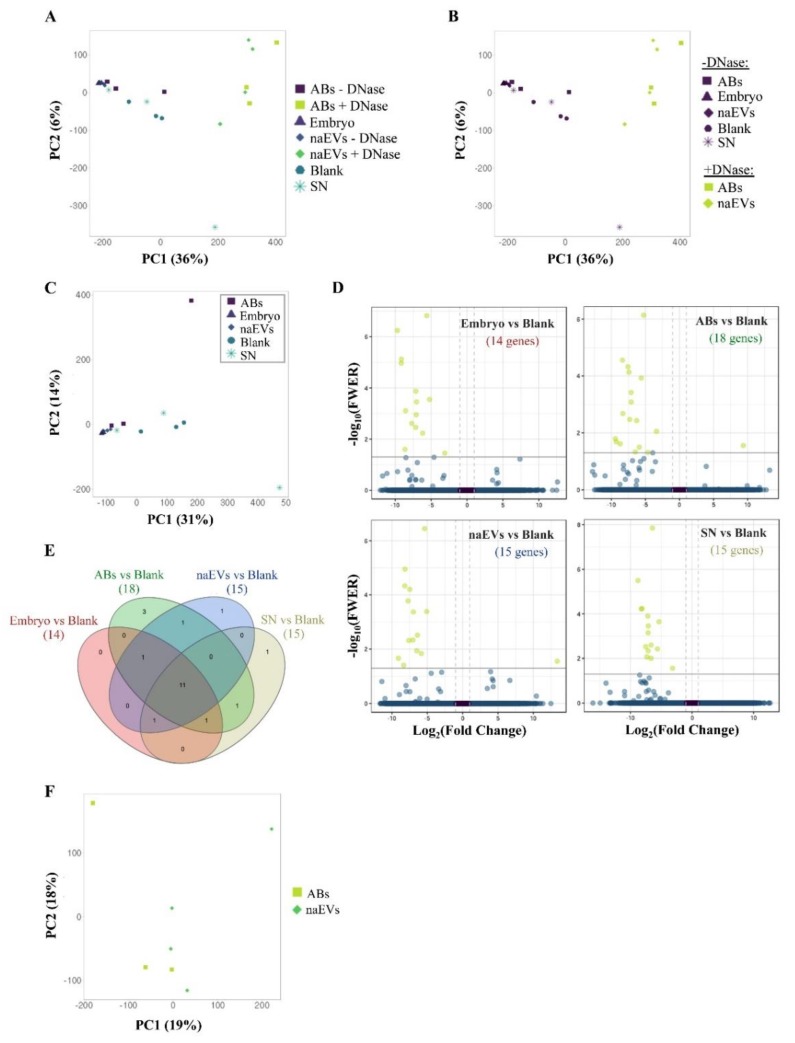
Analysis of the DNA produced and released by the murine embryo to the culture media. (**A**) PCA showing similarity between samples using normalized counts of 27,207 genomic features of the DNase treated and untreated EVs fractions produced by the embryo, the culture media as a negative control (blank), the embryo itself as a positive control, and the SN as an EVs free fraction. (**B**) PCA showing sample distribution by treatment showing separated clusters by treatment. (**C**) PCA of separate analysis using DNase untreated samples, including embryo, Abs, naEVs, SN, and blank control (normalized counts of 26,997 genomic features). PCA shows the similarity between fractions and replicates. (**D**) Volcano plots showing differential enriched genes in the studied fractions (embryo, ABs, naEVs, or SN) compared to the blank control. Negative FC values (left) indicate genes that are enriched in each fraction with respect to the blank medium, while positive values (right) correspond to genes enriched in the blank medium compared to the studied sample. The number of differentially enriched genes is shown in brackets for each comparison. (**E**) Venn diagram showing common and specific enriched genes in pairwise comparisons. (**F**) PCA of DNase treated replicates showed no differences between DNA content in the treated vesicle subpopulations. Abbreviations: AB, apoptotic bodies; naEVs, smaller in size extracellular vesicles (including microvesicles and exosomes); SN, supernatant.

EVs secreted by the embryo, classified as ABs or naEVs, were sequenced and compared. First, DNase treatment was applied to ABs and naEVs fractions and compared to untreated controls to discard the existence of DNA attached to the external membrane of vesicles that could confound with the real cargo. Genomic DNA from the embryos originating the EVs was used as a positive control, SN after the isolation of EVs from the spent media was included as an EVs depleted fraction and oxygenated blank media (G2-plus) as a negative control. Sequencing coverage and mapping statistics are detailed in Table S1. Sequencing of the DNA from different subpopulations revealed no differences among them (Figure 4A). However, DNase treated samples showed a different read count distribution and were differentiated into a separate cluster (Figure 4B, Figure S1), suggesting the existence of external DNA attached to the membrane of vesicles. Paired comparisons of untreated samples showed no statistical differences between the DNA regions observed in ABs and naEVs fractions, neither with the embryo genomic DNA or the EV-depleted SN, but all of them showed significant enrichment in a reduced number of gene sequences compared to the blank media (Figure 4C,D). Eleven of these differential sequences were commonly enriched in all the embryonic fractions against the blank, but no differences among the spent media derived fractions were observed. The complete list of enriched sequences is shown in Table 1 and Tables S2–S5.

DNase treated ABs and naEVs were compared to evaluate their DNA cargo after removal of external DNA. PCA analysis did not cluster separately for both populations and differential enrichment analysis did not evidence any result either (Figure 4F). The absence of differences after DNase treatment may be due to the scarce amount of DNA remaining after treatment, which resulted in poor sequencing outputs (Figure S1, panel C).

Considering these results, the culture media itself was assessed to test whether it could contain contaminating DNA that aligns to the murine genome, masking the results. To evaluate this effect, the reads were aligned to both human and murine genomes. Approximately, 80% of the reads from all the samples, including unused blank media, were able to map both human and murine reference genomes, while only 20% of the reads could be uniquely matched to the murine genome (Figure S2). This fact constituted an important hindrance for the analysis of murine-derived DNA in samples with reduced input and made it impossible to get complete DNA enrichment analyses.

**Figure 5:**
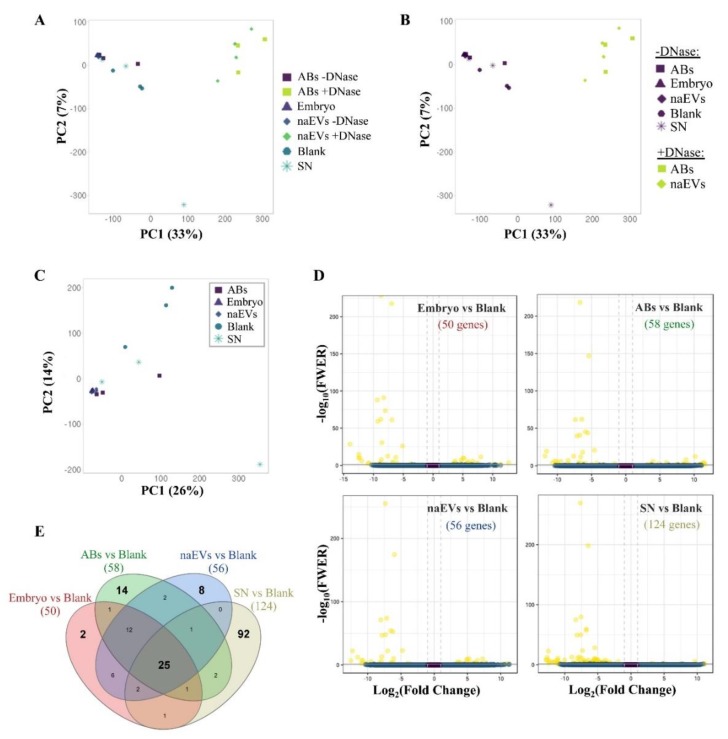
Analysis of murine-specific DNA sequences secreted by the embryo to the culture media. Study of DNA sequences uniquely mapping to Mus musculus reference genome secreted by the murine embryo to the spent media in EVs or as a free form. (**A**) PCA showing similarity between samples using normalized counts of 27,970 genomic features after filtering out human-homologous reads. (**B**) PCA grouping samples by DNase treatment. (**C**) Separate PCA of DNase untreated samples using normalized counts of 27,824 genomic features after filtering out human-homologous reads. (**D**) Volcano plots showing differential enriched genes in the studied fractions (embryo, ABs, naEVs, or SN) compared to the blank control. Negative FC values (left) indicate genes that are enriched in each fraction with respect to the blank medium, while positive values (right) correspond to genes enriched in the blank medium compared to the studied sample. The number of differentially enriched genes is shown in brackets for each comparison. (**E**) Venn diagram showing common and specific enriched genes in pairwise comparisons. Abbreviations: AB, apoptotic bodies; naEVs, smaller in size extracellular vesicles (including microvesicles and exosomes); SN, supernatant.

In this context, those DNA sequences that uniquely map the murine genome were analyzed and only those comparisons of murine DNA isolated from embryos or EVs compared to the blank negative controls were considered. PCA analysis of murine-unique sequences showed again a wide dispersion of the different fractions analyzed (Figure 5A) and only DNase treated versus untreated samples clustered separately (Figure 5B and Figure S1, panels D–F). No major differences in DNA regions were found among DNase untreated fractions (Figure 5C). However, a higher amount of gene sequences was found in all the fractions compared to the blank media (Figure 5D) which only showed artefactual mapping to murine DNA (Figure S3). In this case, 169 gene sequences representing all murine chromosomes were found in the different samples studied (embryo, ABs, naEVs, or SN) compared to the blank, while 25 of them were common to all the comparisons. The individual assessment of each fraction compared to the blank rendered a total of 2, 14, 8, and 92 genes enriched in embryo, ABs, naEVs, and SN, respectively. (Figure 5E, Tables S6–S9). Interestingly, the enriched genes found in vesicles were more similar to the DNA found in the embryo, while the SN showed a different pattern of enriched genes compared to embryo, EVs, and blank media (Figure 5E and Table S10).

## 4. Discussion

The production and secretion of EVs by the embryo have been reported in different species from bovine [39], pig [40], mouse [41], and human [3]. Although different size ranges have been demonstrated among species, all shared phenotypic markers such as CD63 and CD9 [42]. The achievement of blastocyst/hatching stage is key for the release of EVs to the extracellular medium [43]. In humans, the presence of EVs in conditioned media of in vitro cultured human embryos from day three to day five has been previously described as well as their uptake by the maternal side [3]. A similar event occurs in the sheep model, where EVs coming from the conceptus were observed to be internalized by the uterine epithelia, but not in other maternal tissues [24]. Also, of importance is the delivery of EVs between embryo compartments. Particularly, MVs have been observed to be produced by the inner cell mass reaching the trophectoderm, thus promoting its migration and implantation abilities [4]. Our results showed oocytes and embryos in different developmental stages with MVB produced by the blastomeres, as well as secreted vesicles of different sizes (Figure 1A–D). Of note, the existence of large vesicles with complex structures and contents in the intercellular space of the developing embryos was reported, which is in line with Desrochers’ group observations (Figure 1C, D). NTA allows a precise measurement and quantification of EVs by their light scattering properties measured directly by a camera. Results showed a polydisperse population with varying sizes (Figure 2A), which were confirmed by deposition, staining, and TEM visualization of the vesicles (Figure 2B). Regarding molecular markers, immunogold staining of ultrathin cuts from EVs produced by the embryos showed the presence of CD63 and CD9 in the EVs, but also of ARF6, a GTP-binding protein involved in MVs synthesis through the phospholipase D pathway [38]. Importantly, gold labelling also revealed the presence of DNA cargo within these EVs (Figure 3). All-in-all, this initial analysis served us to confirm the production of varying in-size/phenotype EVs by the murine embryos to the culture media, and to associate them with EXOs and MVs markers as well as with DNA cargo. However, the quantification of positive vesicles may be impacted by the method used for the isolation of such vesicles. As high recovery methods may result in a great population of vesicles with low purity showing a low percentage of positive vesicles, while methods that yield a very pure population of vesicles may present a higher degree of positivity while missing other vesicular subtypes that may present a different phenotype. In this work, EVs were recovered using high-speed differential ultracentrifugation after intermediate speed apoptotic bodies removal and washes. This is considered an intermediate recovery and intermediate specificity method [44]. Nonetheless, positivity in our experimental set-up is only descriptive as we analyzed immunogold labelling on ultrathin sections, and only a small representation of the total vesicle fraction has been stained and visualized for this purpose.

Different molecules can be analyzed from the spent media with potential different diagnostic usefulness, including RNA [43] and DNA, both nuclear and mitochondrial [45]. DNA content of EVs is currently a developing field of research. Although different studies coincide in that embryo material in the spent medium may be greatly masked by maternal contamination coming from cumulus cells, its putative clinical usefulness has already been proposed for embryo chromosomal non-invasive diagnosis [43,46,47,48,49,50]. DNA secreted by cells can be single or double stranded at the extracellular medium in its free form, but also externally stuck or included in EVs where it is protected from the enzymes present in the medium [51]. Also, it has been reported that different subpopulations of EVs contain different DNA cargo [16], including mtDNA [52]. It was even observed that EVs’ DNA can be transported into target cells in either the cytoplasm or nuclei [53] where it serves a function [54]. Nevertheless, there is not much information about the DNA content of EVs produced by preimplantation embryos in culture. A recent study reported the presence of DNA in embryo-derived EVs by flow cytometry, associating higher concentrations of DNA-containing EVs with higher rates of implantation failure after embryo transfer. Nevertheless, this may be explained by the fact that these DNA-containing EVs were mainly apoptotic bodies, thus reflecting poor embryo quality [55]. To our knowledge, there are still no works addressing the potential diagnostic value of embryo derived EVs for the detection of DNA mutations/polymorphisms in the cells of origin, but some works in different biofluids, such as blood, urine, or seminal fluid, have done so, highlighting EVs as potential biomarkers for pathological mutations [56]. In order to go one step further into the analysis of the DNA secreted by the preimplantation embryo, the present study analyzes whether any specific cargo was transported inside the EVs released to the spent media during early embryo development.

Pairwise comparisons of embryo fractions—consisting of full-embryos, ABs, MVs, EXOs and SN—against blank media were performed. Differentially enriched genes were observed in all the fractions analyzed and compared to the blank media (Figure 4), although the number of enriched genes was lower than expected. We hypothesized that the small differences found in the DNA enrichment analysis could be influenced by genetic material present in the blank control. G2-plus is a commercial medium supplemented with human serum albumin, which is a blood derived molecule and so it may drag residual DNA from the original source. The possibility of human–mouse cross-species mapping is not trite since 90% of the mouse and human genomes can be partitioned into corresponding regions of conserved synteny. At the nucleotide level, approximately 40% of the human genome, likely representing most of the orthologous sequences, can be aligned to the mouse genome. Around 80% of the mouse genes have a single identifiable orthologue in the human, while only 1% does not have a known homologue [57]. Our hypothesis was confirmed with 80% of the total reads corresponding to blank media aligning both the human and the murine genomes (Figure S2). The analysis of murine-specific DNA sequences in the blanks evidenced poor DNA size and quality and supported the consideration of these samples as background noise (Figure S3). Under this situation, human-homologous reads were filtered out and pairwise comparisons with murine-specific reads were recalculated (Figure 5). New comparisons provided richer results and even a trend was observed showing a high similarity between EVs and embryo DNA regions, while the SN presented a differential DNA pattern. This would introduce the concept of two differentiated embryo DNA sources: vesicular and cell-free DNA, deserving future analysis. Far from intending to establish any functional conclusion and considering the technical limitations of the data analysis, the results presented herein demonstrate the possibility of detecting specific DNA sequences secreted by the murine embryo over the inherent masking of blank medium and point at a differential DNA profile between EVs and SN, which may be of importance and should be further investigated.

Because DNA potentially stuck to the external surface of EVs may influence the results of the sequencing analysis, paired fractions of ABs and naEVs populations were treated with DNase prior to sequencing. Despite having control for human-homologous sequences effect, no statistically significant genes or DNA regions were obtained when comparing specific subtypes of EVs (ABs and naEVs) after DNase treatment. This may be due to the scarce DNA material left for sequencing after the harsh DNase treatment. Further studies starting from a higher number of vesicles and/or using defined embryo culture media might shed light on this point.

## 5. Conclusions

Our results demonstrate the existence of EVs produced by the murine oocyte and embryo throughout embryo development. These EVs are secreted to the extracellular space, and can be found in the intercellular space, the blastocoel or even in the spent culture media, after crossing the zona pellucida. The analysis of the different EVs collected from blastocyst spent media showed the presence of DNA representing the full murine genome, with no differences in DNA cargos between the different fractions (ABs and naEVs). Also, vesicle-free DNA was found in the EVs-depleted supernatant of embryo culture. These findings demonstrate that embryo DNA is randomly secreted to the spent culture medium under physiological conditions without the need of aneuploidy or apoptotic events. Furthermore, our results reinforce the relevance of cellular communication between the conceptus and maternal endometrium during implantation and to support the feasibility of non-invasive testing of pre-implantation embryos.

## Figures and Tables

**Table 1:** Genes enriched in the comparison of the different DNase untreated samples (embryo, ABs, naEVs and SN) against the blank culture medium.

Gene ID	Chr	Embryo vs. Blank		ABs vs. Blank		naEVs vs. Blank		SN vs. Blank	
		log(FC)	FWER	log(FC)	FWER	log(FC)	FWER	log(FC)	FWER
*2610005L07Rik*	8	7.04	3.50 × 10^4^	7.10	3.79 × 10^4^	6.97	4.26 × 10^4^	7.11	3.48 × 10^4^
*Gm10715*	9	9.17	1.08 × 10^5^	7.09	8.17 × 10^4^	7.71	1.67 × 10^4^	8.24	6.06 × 10^5^
*Gm10717*	9	7.71	2.44 × 10^3^	5.89	3.18 × 10^2^	6.50	1.15 × 10^2^	7.13	4.57 × 10^3^
*Gm13822*	5	7.14	1.35 × 10^4^	7.42	7.34 × 10^5^	7.47	6.25 × 10^5^	7.18	1.27 × 10^4^
*Gm17535*	9	9.71	5.68 × 10^7^	7.57	4.76 × 10^5^	8.15	1.11 × 10^5^	8.77	3.19 × 10^6^
*Gm26624*	4	5.22	2.83 × 10^4^	5.62	1.17 × 10^4^	5.09	4.09 × 10^4^	5.37	2.29 × 10^4^
*Gm26804*	8	7.15	3.47 × 10^3^	7.30	3.35 × 10^3^	7.03	4.63 × 10^3^	6.66	9.83 × 10^3^
*Gm7120*	13	6.20	5.84 × 10^3^	6.29	3.69 × 10^3^	6.39	3.04 × 10^3^	7.14	7.15 × 10^4^
*Pisd-ps1*	11	9.14	7.51 × 10^6^	8.38	2.77 × 10^5^	8.17	4.65 × 10^5^	8.12	5.83 × 10^5^
*Pisd-ps2*	17	8.56	7.81 × 10^4^	8.31	2.08 × 10^3^	7.61	4.78 × 10^3^	7.34	8.52 × 10^3^
*Sfi1*	11	5.61	1.49 × 10^7^	5.26	7.32 × 10^7^	5.38	3.56 × 10^7^	6.42	1.40 × 10^8^
*C230088H06Rik*	4	3.10	3.53 × 10^2^	3.38	8.85 × 10^3^			3.18	2.73 × 10^2^
*Gm10720*	9	7.09	1.11 × 10^3^			5.84	1.44 × 10^2^	6.64	2.54 × 10^3^
*Gm14412*	2	8.66	2.51 × 10^2^	8.70	2.41 × 10^2^	8.35	3.86 × 10^2^		
*C130026I21Rik*	1			4.64	4.84 × 10^2^			5.61	3.80 × 10^3^
*Gm13251*	4			9.41	1.60 × 10^2^	9.08	2.15 × 10^2^		
*Cd2bp2*	7			−9.38	2.77 × 10^2^				
*Fbxw18*	9							7.53	2.98 × 10^3^
*Gm10306*	4					−13.38	2.75 × 10^2^		
*Gm13154*	4			6.50	4.66 × 10^2^				
*Leprot*	4			9.29	2.04 × 10^2^				

Abbreviations: ABs, Apoptotic Bodies; Chr, Chromosome; FC, Fold Change; FWER, Family-Wise Error Rate; naEVs, smaller in size extracellular vesicles (including microvesicles and exosomes); SN, supernatant.

## Supplementary Materials

The following are available online at https://www.mdpi.com/2073-4425/11/2/203/s1, Figure S1: DNA sequencing read counts and normalization of the different fractions. Plots showing normalization of samples sequencing reads previous (A, B, C) and after (D, E, F) filtering by specific murine genome. (A and D). All samples populations. (B and E) DNase untreated samples. (C and F) DNase treated EVs. Abbreviations: ABs: Apoptotic bodies; CPM, Counts per million; naEVs: non-apoptotic EVs (including microvesicles and exosomes); SN: supernatant; Figure S2: Mouse-Human cross-mapping. Normalized plot showing the percentages of reads for each sample able to map both to the human and murine reference genomes (purple) and those mapping uniquely to the murine genome (yellow). Abbreviations: ABs: Apoptotic bodies; naEVs: non-apoptotic EVs (including microvesicles and exosomes); SN: supernatant; Figure S3: Quality control of murine specific DNA sequences. (A) Murine genomic sequences detected in blank samples showed poor mapping quality (MAPQ score) compared to embryo-derived samples. Murine genes differentially enriched in the experimental setting showed poor mapping quality (B) and insert insert size (C) in blank samples compared to embryo-derived fractions; Table S1: DNA sequencing reads, and mapping statistics; Table S2: Sequencing analysis data for the enriched genes in the comparison Embryo vs blank control for total gene sequences; Table S3: Sequencing analysis data for the enriched genes in the comparison ABs vs blank control for total gene sequences; Table S4: Sequencing analysis data for the enriched genes in the comparison naEVs vs blank control for total gene sequences; Table S5: Sequencing analysis data for the enriched genes in the comparison SN vs blank control for total gene sequences; Table S6: Sequencing analysis data for the enriched genes in the comparison Embryo vs blank control for gene sequences uniquely binding the Mus musculus reference genome; Table S7: Sequencing analysis data for the enriched genes in the comparison ABs vs blank control for gene sequences uniquely binding the Mus musculus reference genome; Table S8: Sequencing analysis data for the enriched genes in the comparison naEVs vs blank control for gene sequences uniquely binding the Mus musculus reference genome; Table S9: Sequencing analysis data for the enriched genes in the comparison SN vs blank control for gene sequences uniquely binding the Mus musculus reference genome; Table S10: Gene sequences uniquely mapping to the Mus musculus reference genome enriched in the comparisons of the different DNase untreated samples (embryo genomic DNA, ABs, naEVs and SN) vs the blank control. Abbreviations: ABs, Apoptotic Bodies; Chr, Chromosome; FC, FoldChange; FWER, Family-Wise Error Rate; naEVs, non-apoptotic EVs; SN, Supernatant.
