# Supplementary material for: Identification and Characterization of Extracellular Vesicles and Its DNA Cargo Secreted During Murine Embryo Development
Source: Genes (Basel). 2020 Feb 17;11(2):203. doi: 10.3390/genes11020203 (PMC7074575; doi:10.3390/genes11020203)
Supplement: Supplementary file 1 [file genes-11-00203-s001.zip › Table S1.pdf]

Table S1. DNA sequencing reads, and mapping statistics.

Sample	Total reads	Unmapped reads (%)	q10 reads (%)	Duplicate reads (%)	Valid reads (%)
Embryo -DNase Set A	54,274,197	180,665 (0.33)	24,917,798 (45.91)	7,167,123 (13.21)	22,008,611 (40.55)
Embryo -DNase Set B	35,827,722	101,039 (0.28)	14,724,678 (41.10)	5,238,097 (14.62)	15,763,908 (44.00)
Embryo -DNase Set C	37,511,254	108,280 (0.29)	14,550,852 (38.79)	4,294,532 (11.45)	18,557,590 (49.47)
Embryo -DNase Set D	28,362,627	101,760 (0.36)	7,028,758 (24.78)	3,037,951 (10.71)	18,194,158 (64.15)
AB -DNase Set A	60,941,941	894,888 (1.47)	10,025,278 (16.45)	7,022,766 (11.52)	42,999,009 (70.56)
AB -DNase Set B	50,857,885	166,853 (0.33)	8,514,415 (16.74)	2,951,505 (5.80)	39,225,112 (77.13)
AB -DNase Set D	50,051,241	183,887 (0.37)	9,322,997 (18.63)	3,598,577 (7.19)	36,945,780 (73.82)
AB +DNase Set A	48,055,108	18,715,589 (38.95)	15,347,029 (31.94)	9,374,708 (19.51)	4,617,782 (9.61)
AB +DNase Set B	41,517,087	13,821,014 (33.29)	11,139,708 (26.83)	8,385,028 (20.20)	8,171,337 (19.68)
AB +DNase Set D	54,989,754	4,841,168 (8.80)	22,533,188 (40.98)	24,229,269 (44.06)	3,386,129 (6.16)
naEVs -DNase Set A	55,700,778	168,107 (0.30)	19,712,479 (35.39)	4,931,459 (8.85)	30,888,733 (55.45)
naEVs -DNase Set B	26,138,898	102,238 (0.39)	7,610,236 (29.11)	1,618,270 (6.19)	16,808,154 (64.30)
naEVs -DNase Set C	29,684,788	81,242 (0.27)	5,839,112 (19.67)	2,107,135 (7.10)	21,657,299 (72.96)
naEVs -DNase Set D	45,018,553	163,213 (0.36)	9,205,664 (20.45)	3,071,051 (6.82)	32,578,625 (72.37)
naEVs +DNase Set A	63,060,670	3,669,169 (5.82)	21,735,122 (34.47)	22,812,610 (36.18)	14,843,769 (23.54)
naEVs +DNase Set B	37,790,010	9,618,074 (25.42)	13,190,374 (34.90)	7,502,293 (19.85)	7,479,269 (19.79)
naEVs +DNase Set C	30,470,420	4,679,611 (15.36)	10,943,879 (35.92)	9,005,929 (29.56)	5,841,001 (19.17)
naEVs +DNase Set D	38,060,061	7,895,316 (20.74)	12,140,036 (31.90)	11,852,748 (31.14)	6,171,961 (16.22)
Blank -Dnase Set A	48,626,830	22,748,470 (46.78)	13,201,966 (27.15)	3,447,837 (7.09)	9,228,557 (18.98)
Blank -Dnase Set B	33,280,407	16,576,757 (49.81)	8,740,996 (26.26)	1,828,273 (5.49)	6,134,381 (18.43)
Blank -Dnase Set D	53,970,000	24,900,066 (46.14)	14,234,067 (26.37)	2,367,844 (4.39)	12,468,023 (23.10)
SN -DNase Set A	54,334,141	6,957,272 (12.80)	11,058,495 (20.35)	12,583,159 (23.16)	23,735,215 (43.68)
SN -DNase Set B	23,756,570	1,109,633 (4.67)	4,812,808 (20.26)	1,512,310 (6.37)	16,321,819 (68.70)
SN -DNase Set D	43,776,010	2,176,044 (4.97)	13,624,551 (31.12)	4,165,205 (9.51)	23,810,210 (54.39)
