# Supplementary material for: Identification and Characterization of Extracellular Vesicles and Its DNA Cargo Secreted During Murine Embryo Development
Source: Genes (Basel). 2020 Feb 17;11(2):203. doi: 10.3390/genes11020203 (PMC7074575; doi:10.3390/genes11020203)
Supplement: Supplementary file 1 [file genes-11-00203-s001.zip › Table S10.pdf]

Table S10. Gene sequences uniquely mapping to the *Mus musculus* reference genome enriched in the comparisons of the different DNase untreated samples (embryo genomic DNA, ABs, naEVs and SN) vs the blank control. Abbreviations: ABs, Apoptotic Bodies; Chr, Chromosome; FC, Fold Change; FWER, Family-Wise Error Rate; naEVs, non-apoptotic EVs; SN, Supernatant.

		Embryo vs Blank		ABs vs Blank		naEVs vs Blank		SN vs Blank	
**Gene ID**	**Chr**	**log(FC)**	**FWER**	**log(FC)**	**FWER**	**log(FC)**	**FWER**	**log(FC)**	**FWER**
2610005L07Rik	8	6.81	3.65E-05	6.85	5.45E-05	6.48	1.57E-04	5.98	1.31E-03
C1qb	4	-7.11	1.64E-03	-7.61	4.30E-03	-6.52	2.37E-02	-9.62	1.98E-06
Cd2bp2	7	-8.01	6.88E-12	-10.17	5.02E-13	-7.27	1.94E-10	-4.89	9.23E-03
Cd5l	3	8.80	8.68E-05	9.48	1.76E-06	9.50	2.09E-06	12.68	4.63E-13
Gm10715	9	12.48	9.23E-11	10.07	3.29E-06	10.72	1.53E-07	11.03	5.43E-08
Gm10717	9	9.35	1.08E-88	7.35	1.96E-62	8.02	5.00E-72	8.33	1.25E-75
Gm10718	9	8.01	3.42E-74	5.90	5.67E-46	6.53	4.88E-55	6.77	2.76E-58
Gm10719	9	8.59	4.44E-31	6.31	1.21E-18	7.02	6.72E-23	7.33	2.08E-24
Gm10720	9	12.31	1.21E-10	10.34	7.79E-07	10.92	4.95E-08	11.17	2.14E-08
Gm10722	9	9.26	8.22E-60	7.13	1.70E-40	7.85	1.78E-47	8.16	5.02E-50
Gm10800	2	6.94	2.12E-218	5.40	1.63E-147	5.99	2.39E-175	6.46	5.68E-199
Gm10801	2	6.90	7.55E-62	5.57	1.80E-44	6.23	2.54E-53	6.74	3.26E-60
Gm11168	9	8.76	1.78E-62	6.69	1.88E-41	7.32	1.82E-48	7.58	1.24E-50
Gm11399	9	10.78	5.83E-04	9.96	7.98E-03	10.12	4.01E-03	10.01	7.77E-03
Gm13822	5	6.07	3.06E-04	5.87	7.55E-04	6.12	2.11E-04	5.87	7.85E-04
Gm17535	5	13.92	3.87E-29	11.58	3.90E-20	12.28	5.09E-23	12.48	1.48E-23
Gm21738	5	8.27	1.23E-91	6.44	9.43E-63	7.14	1.29E-74	7.55	2.02E-80
Gm26624	5	6.23	2.52E-11	6.59	2.88E-12	6.12	5.62E-11	5.60	4.51E-09
Gm26804	8	6.65	3.62E-04	6.85	2.99E-04	6.42	9.35E-04	5.44	3.95E-02
Gm26870	8	8.75	0.00E+00	6.68	4.16E-219	7.34	5.78E-256	7.65	4.43E-270
Gm7120	8	7.62	5.52E-13	7.62	2.89E-13	7.90	5.79E-14	7.78	1.40E-13
Pisd-ps1	8	12.44	2.22E-15	11.81	1.51E-13	11.52	6.05E-13	11.03	1.52E-11
Pisd-ps2	9	8.68	2.13E-08	8.43	1.51E-07	7.98	6.27E-07	7.21	2.48E-05
Sfi1	9	5.13	1.32E-26	4.59	3.11E-21	4.79	1.59E-23	5.46	6.46E-29
Ttc39c	18	-3.71	2.89E-06	-4.00	1.66E-06	-3.62	9.73E-06	-3.24	4.26E-03
Bhlhe22	3	-10.56	3.18E-02	-11.19	5.76E-03			-11.16	3.93E-02
Crybb3	5	-6.02	1.27E-03	-6.13	6.10E-03	-6.24	5.41E-04		
Dll3	7	-5.83	5.37E-06	-5.93	5.25E-05	-6.49	6.05E-08		
Dlst	12	-4.68	8.90E-04	-4.79	1.65E-03	-4.35	1.38E-02		
Echdc2	4	7.83	2.90E-02			7.90	2.71E-02	12.83	9.46E-09
Gm13251	4	9.93	3.24E-02	10.28	1.66E-02	10.35	1.22E-02		
Gm14838	X	-11.36	2.58E-02	-11.62	7.10E-03	-11.09	1.77E-02		
Gm15544	7			8.47	2.29E-03	8.27	2.40E-03	12.72	2.92E-09
Gm19557	19	10.90	4.04E-03			10.94	1.17E-02	12.34	2.29E-07
Gm21847	5	3.76	3.85E-03	4.24	2.80E-04	3.96	1.19E-03		
Gm21967	13	11.97	9.58E-07	10.69	1.16E-04	12.55	1.42E-07		
Gm26675	16	-5.16	1.91E-10	-3.21	4.27E-02	-5.26	1.87E-10		
Klra2	6	-5.65	9.79E-04	-8.81	1.90E-08	-5.60	1.85E-03		
Naa60	16	-4.72	7.67E-08	-3.66	2.87E-03	-4.89	3.48E-09		
Pcx	19	-3.57	2.87E-04	-3.07	4.06E-02	-3.53	3.18E-04		
Zfp597	16	-5.04	3.18E-10	-3.22	2.64E-02	-4.95	6.95E-10		
Cdh23	10			1.65	1.00E-03			1.95	3.98E-06
Gm16028	1	9.35	2.41E-02					11.09	8.91E-04
Lhx5	5	-6.38	4.90E-02	-8.79	2.78E-03				
Myl1	1	-6.62	3.02E-06			-5.14	1.19E-02		
Nol12	15			-9.23	4.98E-03	-6.78	4.84E-02		
Oas1h	5			9.82	6.87E-03			11.71	3.01E-05
Rps6ka4	19	-7.54	6.02E-04			-7.02	2.03E-02		
Sgcb	5	-5.90	3.11E-03			-5.92	3.54E-03		

Socs7	11	-3.75	2.94E-03			-3.64	7.49E-03		
Stat4	1	-3.78	4.71E-03			-3.95	6.37E-04		
Tas2r116	6			-11.41	5.32E-03	-10.92	1.10E-02		
Wdr33	18	-3.21	9.04E-03			-2.96	3.89E-02		
1700001L05Rik	15							10.69	1.65E-02
1700003F17Rik	11							6.99	2.91E-02
3010026O09Rik	11							8.47	9.13E-04
4632404H12Rik	3			-10.91	9.06E-03				
4930525M21RIK	X							11.01	1.12E-02
4932415M13Rik	17							11.71	5.95E-05
4932442E05Rik	10			-11.44	5.31E-05				
Aatk	11							4.12	7.46E-04
Abcc6	7							5.10	2.77E-02
Acbd5	2							7.09	9.15E-05
Actr3b	5							5.70	1.23E-07
Aftph	11							4.04	1.45E-05
Aim1l	4							4.93	4.91E-02
Aldh1a3	7							4.87	5.02E-05
Alox8	11							7.90	6.11E-04
Ank1	8			3.14	1.86E-02				
Atp5sl	7							12.45	3.15E-03
Azi1	11							6.53	2.97E-05
Babam1	8							4.92	4.33E-03
BC025920	10							6.77	1.85E-03
Cacng3	7							3.77	3.15E-02
Calcrl	2							-5.17	3.59E-02
Cass4	2							3.79	5.81E-04
Ccdc40	11							5.50	3.02E-11
Ccdc74a	16			-10.96	1.95E-02				
Chat	14							4.15	3.34E-02
Chchd4	6							6.55	1.76E-03
Chrna2	14							5.51	2.50E-02
Col5a3	9							5.44	5.51E-06
Cope	8							8.76	3.32E-04
Cpb1	3							5.31	9.30E-08
Cpn1	19							5.57	8.34E-03
Creb3l2	6							3.38	1.65E-02
Crlf3	11							7.25	9.13E-05
Crygn	5			-7.42	1.59E-02				
Cyp2b13	7			4.97	7.54E-03				
D030025P21Rik	12	-12.69	3.41E-04						
Dclk3	9							4.42	3.59E-04
Dmgdh	13							4.65	7.26E-04
Etnk1	6					-3.88	3.02E-02		
Exosc10	4							5.65	9.02E-03
Fa2h	8							6.05	6.02E-05
Fam21	6							5.88	4.05E-05
Fbxl14	6			-11.28	3.55E-02				
Fbxl3	14			-8.45	8.72E-03				
Flrt1	19							10.03	8.19E-09
G6pc	11							11.14	5.47E-03
G6pc3	11							-11.04	1.32E-02
Gal3st3	19							5.32	4.46E-02
Galns	8							5.72	1.08E-04
Galr2	11					-5.95	3.27E-03		
Gas6	8							8.95	1.43E-13
Gm11033	8							2.91	1.04E-02

Gm11998	11							7.92	3.94E-06
Gm12802	4					-7.75	2.43E-04		
Gm13373	2							11.70	3.98E-05
Gm14040	2			6.29	6.73E-04				
Gm15199	10							5.95	1.28E-04
Gm16630	6							3.66	1.69E-02
Gm16638	7							4.97	1.21E-04
Gm16976	14							4.27	2.26E-04
Gm17344	8							8.88	3.27E-02
Gm21747	18							12.35	8.47E-06
Gm26896	10							8.52	4.73E-03
Gpnmb	6							5.49	2.88E-06
Grik3	4							3.33	3.37E-05
Hgfac	5							6.70	1.79E-02
Hmgn2	4					-10.32	3.17E-02		
Hook2	8							5.89	1.10E-02
Hps1	19					-4.31	1.70E-02		
Hsd17b3	13							5.73	7.66E-04
Il17rc	6							7.00	1.31E-03
Inf2	12					-5.03	3.74E-02		
Itgb5	16							3.40	2.34E-02
Kif13b	14							3.23	2.68E-02
Leprot	4			8.03	4.50E-02				
Ltb4r2	14	-10.04	3.24E-02						
Mroh2b	15							4.60	2.75E-02
Mroh4	15							5.40	3.03E-08
Mug1	6							5.03	1.79E-03
Ndufb8	19					-7.31	8.76E-03		
Nxph4	10							8.71	7.42E-03
Obfc1	19							4.49	1.81E-03
Parp1	1							8.08	5.83E-12
Pet112	3							5.15	4.70E-04
Pip5k1c	10							4.17	3.67E-02
Pitx1	13			-8.59	2.56E-02				
Pkd1l1	11							4.99	3.43E-09
Ppm1h	10							3.02	2.22E-03
Ppp2r5a	1					-5.17	5.10E-03		
Prkch	12							3.23	1.60E-02
Prkci	3							4.80	6.42E-04
Prkcz	4							5.36	3.35E-10
Pura	18							6.96	1.13E-05
Rnf180	13							4.38	3.34E-05
Ror2	13							3.85	1.70E-08
Scarb1	5							4.76	2.31E-02
Slc22a8	19							6.15	2.09E-02
Snx16	3							4.98	4.50E-02
Socs3	11			-10.60	1.13E-02				
Tas1r2	4							5.71	2.49E-03
Tceanc2	4							5.11	4.08E-09
Tfap2a	13			-8.34	2.28E-03				
Tle1	4							4.07	4.57E-03
Tlr5	1							7.09	5.17E-08
Tm7sf2	19							6.18	2.82E-02
Tmc3	7							4.86	1.42E-05
Tmem63a	1							4.64	9.31E-03
Tmie	9							7.43	1.77E-11
Tspan3	9							6.46	1.99E-08

Tspan6	X							-7.47	1.90E-02
Vezt	10							4.46	1.40E-03
Vmn2r52	7			-11.21	2.98E-04				
Zfp426	9							6.87	1.04E-05
Zfp57	17							10.55	4.73E-02
