# Supplementary material for: Identification and Characterization of Extracellular Vesicles and Its DNA Cargo Secreted During Murine Embryo Development
Source: Genes (Basel). 2020 Feb 17;11(2):203. doi: 10.3390/genes11020203 (PMC7074575; doi:10.3390/genes11020203)
Supplement: Supplementary file 1 [file genes-11-00203-s001.zip › Table S2.pdf]

Table S2. Sequencing analysis data for the enriched genes in the comparison Embryo vs blank control for total gene sequences.

id	chr	start	end	gene	logFC	logCPM	FWER
chr11_3125904_3131004	chr11	3125904	3131004	*Pisd-ps1*	9,14344287	7,39725547	7,51079E-06
chr11_3131850_3193463	chr11	3131850	3193463	*Sfi1*	5,61433566	10,22435	1,49206E-07
chr13_119487941_119610458	chr13	1,19E+08	1,2E+08	*Gm7120*	6,19544234	7,26337718	0,005840259
chr17_3076578_3084183	chr17	3076578	3084183	*Pisd-ps2*	8,56004983	6,51527195	0,000781297
chr2_177314520_177324307	chr2	1,77E+08	1,77E+08	*Gm14412*	8,65659082	5,31266606	0,025122778
chr4_147018235_147492201	chr4	1,47E+08	1,47E+08	*C230088H06Rik*	3,10307501	8,92240706	0,035323838
chr4_147136278_147457019	chr4	1,47E+08	1,47E+08	*Gm26624*	5,22356555	8,42474861	0,000282673
chr5_114917318_114923277	chr5	1,15E+08	1,15E+08	*Gm13822*	7,14007375	6,68846706	0,00013457
chr8_20267774_20424814	chr8	20267774	20424814	*2610005L07Rik*	7,04452224	6,89615229	0,000350043
chr8_20292477_20420781	chr8	20292477	20420781	*Gm26804*	7,15069283	6,50456744	0,003469338
chr9_3015654_3017210	chr9	3015654	3017210	*Gm10720*	7,08801769	7,89732859	0,001110643
chr9_3025417_3033289	chr9	3025417	3033289	*Gm10717*	7,70653357	10,8861307	0,002442111
chr9_3034599_3035805	chr9	3034599	3035805	*Gm17535*	9,71364358	7,80542113	5,6766E-07
chr9_3036877_3038316	chr9	3036877	3038316	*Gm10715*	9,16978639	7,29750581	1,08229E-05
