# Supplementary material for: Identification and Characterization of Extracellular Vesicles and Its DNA Cargo Secreted During Murine Embryo Development
Source: Genes (Basel). 2020 Feb 17;11(2):203. doi: 10.3390/genes11020203 (PMC7074575; doi:10.3390/genes11020203)
Supplement: Supplementary file 1 [file genes-11-00203-s001.zip › Table S3.pdf]

Table S3. Sequencing analysis data for the enriched genes in the comparison ABs vs blank control for total gene sequences.

id	chr	start	end	gene	logFC	logCPM	FWER
chr11_3125904_3131004	chr11	3125904	3131004	*Pisd-ps1*	8,377409728	7,397255473	2,77181E-05
chr11_3131850_3193463	chr11	3131850	3193463	*Sfi1*	5,263058812	10,22434998	7,32064E-07
chr13_119487941_119610458	chr13	119487941	119610458	*Gm7120*	6,288160646	7,263377184	0,003691524
chr17_3076578_3084183	chr17	3076578	3084183	*Pisd-ps2*	8,308132225	6,515271948	0,002081191
chr1_84992690_85270566	chr1	84992690	85270566	*C130026I21Rik*	4,641247141	7,835852661	0,048388644
chr2_177314520_177324307	chr2	177314520	177324307	*Gm14412*	8,700030176	5,312666059	0,02411207
chr4_101647718_101659364	chr4	101647718	101659364	*Leprot*	9,285428475	5,488775337	0,02044234
chr4_146449023_146470292	chr4	146449023	146470292	*Gm13251*	9,413498047	5,716532392	0,016001444
chr4_147018235_147492201	chr4	147018235	147492201	*C230088H06Rik*	3,380258801	8,922407064	0,008846457
chr4_147136278_147457019	chr4	147136278	147457019	*Gm26624*	5,61626115	8,424748607	0,000117139
chr4_147553277_147585198	chr4	147553277	147585198	*Gm13154*	6,49827767	6,296469044	0,046569157
chr5_114917318_114923277	chr5	114917318	114923277	*Gm13822*	7,420537693	6,688467058	7,34011E-05
chr7_127191660_127196000	chr7	127191660	127196000	*Cd2bp2*	-9,375145172	5,667493084	0,027722358
chr8_20267774_20424814	chr8	20267774	20424814	*2610005L07Rik*	7,095771195	6,896152288	0,000379027
chr8_20292477_20420781	chr8	20292477	20420781	*Gm26804*	7,302874415	6,50456744	0,003349933
chr9_3025417_3033289	chr9	3025417	3033289	*Gm10717*	5,89472124	10,88613073	0,031777456
chr9_3034599_3035805	chr9	3034599	3035805	*Gm17535*	7,566832805	7,805421127	4,76437E-05
chr9_3036877_3038316	chr9	3036877	3038316	*Gm10715*	7,086439359	7,297505812	0,000816605
