# Supplementary material for: Identification and Characterization of Extracellular Vesicles and Its DNA Cargo Secreted During Murine Embryo Development
Source: Genes (Basel). 2020 Feb 17;11(2):203. doi: 10.3390/genes11020203 (PMC7074575; doi:10.3390/genes11020203)
Supplement: Supplementary file 1 [file genes-11-00203-s001.zip › Table S4.pdf]

**Table S4. Sequencing analysis data for the enriched genes in the comparison naEVs vs blank control for total gene sequences.**

| id | chr | start | end | gene | logFC | logCPM | FWER |
|---|---|---|---|---|---|---|---|
| chr11_3125904_3131004 | chr11 | 3125904 | 3131004 | *Pisd-ps1* | 9.172573713 | 7.397255473 | 4.64557E-05 |
| chr11_3131850_3193463 | chr11 | 3131850 | 3193463 | *Sfi1* | 5.379296795 | 10.22434998 | 3.56108E-07 |
| chr13_119487941_119610458 | chr13 | 119487941 | 119610458 | *Gm7120* | 6.38645144 | 7.263377184 | 0.003043154 |
| chr17_3076578_3084183 | chr17 | 3076578 | 3084183 | *Pisd-ps2* | 7.60704978 | 6.515271948 | 0.004782967 |
| chr2_177314520_177324307 | chr2 | 177314520 | 177324307 | *Gm14412* | 8.346407844 | 5.312666059 | 0.038646267 |
| chr4_146449023_146470292 | chr4 | 146449023 | 146470292 | *Gm13251* | 9.078900696 | 5.716532392 | 0.021494435 |
| chr4_147136278_147457019 | chr4 | 147136278 | 147457019 | *Gm26624* | 5.087253628 | 8.424748607 | 0.000408962 |
| chr4_94556546_94557078 | chr4 | 94556546 | 94557078 | *Gm10306* | -13.37921866 | 3.823907265 | 0.027535255 |
| chr5_114917318_114923277 | chr5 | 114917318 | 114923277 | *Gm13822* | 7.469887046 | 6.688467058 | 6.25324E-05 |
| chr8_20267774_20424814 | chr8 | 20267774 | 20424814 | *2610005L07Rik* | 6.972939883 | 6.896152288 | 0.000425689 |
| chr8_20292477_20420781 | chr8 | 20292477 | 20420781 | *Gm26804* | 7.025694476 | 6.50456744 | 0.004627101 |
| chr9_3015654_3017210 | chr9 | 3015654 | 3017210 | *Gm10720* | 5.84286223 | 7.897328595 | 0.01442196 |
| chr9_3025417_3033289 | chr9 | 3025417 | 3033289 | *Gm10717* | 6.495121466 | 10.88613073 | 0.011519888 |
| chr9_3034599_3035805 | chr9 | 3034599 | 3035805 | *Gm17535* | 8.148925239 | 7.805421127 | 1.10621E-05 |
| chr9_3036877_3038316 | chr9 | 3036877 | 3038316 | *Gm10715* | 7.711741567 | 7.297505812 | 0.000166673 |
