# Supplementary material for: Identification and Characterization of Extracellular Vesicles and Its DNA Cargo Secreted During Murine Embryo Development
Source: Genes (Basel). 2020 Feb 17;11(2):203. doi: 10.3390/genes11020203 (PMC7074575; doi:10.3390/genes11020203)
Supplement: Supplementary file 1 [file genes-11-00203-s001.zip › Table S5.pdf]

Table S5. Sequencing analysis data for the enriched genes in the comparison SN vs blank control for total gene sequences.

id	chr	start	end	gene	logFC	logCPM	FWER
chr11_3125904_3131004	chr11	3125904	3131004	*Pisd-ps1*	8,119390347	7,397255473	5,83453E-05
chr11_3131850_3193463	chr11	3131850	3193463	*Sfi1*	6,424795282	10,22434998	1,39943E-08
chr13_119487941_119610458	chr13	119487941	119610458	*Gm7120*	7,136693317	7,263377184	0,000714972
chr17_3076578_3084183	chr17	3076578	3084183	*Pisd-ps2*	7,337428858	6,515271948	0,008524015
chr1_84992690_85270566	chr1	84992690	85270566	*C130026I21Rik*	5,611124255	7,835852661	0,00380244
chr4_147018235_147492201	chr4	147018235	147492201	*C230088H06Rik*	3,177580342	8,922407064	0,027282166
chr4_147136278_147457019	chr4	147136278	147457019	*Gm26624*	5,367707959	8,424748607	0,000228725
chr5_114917318_114923277	chr5	114917318	114923277	*Gm13822*	7,178907453	6,688467058	0,000126946
chr8_20267774_20424814	chr8	20267774	20424814	*2610005L07Rik*	7,110188675	6,896152288	0,000348045
chr8_20292477_20420781	chr8	20292477	20420781	*Gm26804*	6,663357319	6,50456744	0,009825116
chr9_109676734_109702700	chr9	109676734	109702700	*Fbxw18*	7,532741397	6,779255967	0,002975929
chr9_3015654_3017210	chr9	3015654	3017210	*Gm10720*	6,641446263	7,897328595	0,00253916
chr9_3025417_3033289	chr9	3025417	3033289	*Gm10717*	7,13252915	10,88613073	0,004574878
chr9_3034599_3035805	chr9	3034599	3035805	*Gm17535*	8,773454196	7,805421127	3,19138E-06
chr9_3036877_3038316	chr9	3036877	3038316	*Gm10715*	8,24416085	7,297505812	6,05965E-05
