# Supplementary material for: Identification and Characterization of Extracellular Vesicles and Its DNA Cargo Secreted During Murine Embryo Development
Source: Genes (Basel). 2020 Feb 17;11(2):203. doi: 10.3390/genes11020203 (PMC7074575; doi:10.3390/genes11020203)
Supplement: Supplementary file 1 [file genes-11-00203-s001.zip › Table S6.pdf]

**Table S6. Sequencing analysis data for the enriched genes in the comparison Embryo vs blank control for gene sequences uniquely binding the *Mus musculus* reference genome.**

| id | chr | start | end | gene | logFC | logCPM | FWER |
|---|---|---|---|---|---|---|---|
| chr11_3125904_3131004 | chr11 | 3125904 | 3131004 | *Pisd-ps1* | 12,4434361 | 7,546045555 | 2,22088E-15 |
| chr11_3131850_3193463 | chr11 | 3131850 | 3193463 | *Sfi1* | 5,126255729 | 10,26359019 | 1,32183E-26 |
| chr11_3142048_3143363 | chr11 | 3142048 | 3143363 | *Gm11399* | 10,7839565 | 5,885694338 | 0,000583145 |
| chr11_97362551_97398542 | chr11 | 97362551 | 97398542 | *Socs7* | -3,747903091 | 7,700942682 | 0,002937969 |
| chr12_84875769_84878132 | chr12 | 84875769 | 84878132 | *D030025P21Rik* | -12,69387428 | 5,759235308 | 0,000340618 |
| chr12_85110833_85134091 | chr12 | 85110833 | 85134091 | *Dlst* | -4,676679299 | 7,350444007 | 0,000889823 |
| chr13_119487941_119610458 | chr13 | 119487941 | 119610458 | *Gm7120* | 7,617197767 | 8,016567803 | 5,52161E-13 |
| chr13_119606650_119610458 | chr13 | 119606650 | 119610458 | *Gm21967* | 11,97491097 | 6,493150042 | 9,57875E-07 |
| chr14_19415857_19418930 | chr14 | 19415857 | 19418930 | *Gm21738* | 8,273137037 | 11,27633001 | 1,23374E-91 |
| chr14_55761428_55763229 | chr14 | 55761428 | 55763229 | *Ltb4r2* | -10,04299512 | 4,214700737 | 0,032437241 |
| chr1_51987148_52107188 | chr1 | 51987148 | 52107188 | *Stat4* | -3,777095341 | 8,015519032 | 0,004708806 |
| chr16_3858321_3884561 | chr16 | 3858321 | 3884561 | *Zfp597* | -5,036663232 | 8,274119125 | 3,1775E-10 |
| chr16_3862384_3884505 | chr16 | 3862384 | 3884505 | *Gm26675* | -5,164476194 | 8,233242076 | 1,91139E-10 |
| chr16_3872375_3904770 | chr16 | 3872375 | 3904770 | *Naa60* | -4,71828314 | 8,18896789 | 7,66701E-08 |
| chr1_66924295_66945404 | chr1 | 66924295 | 66945404 | *Myl1* | -6,622947525 | 6,768925192 | 3,01875E-06 |
| chr17_3076578_3084183 | chr17 | 3076578 | 3084183 | *Pisd-ps2* | 8,675690394 | 7,019574733 | 2,13369E-08 |
| chr18_12599926_12737050 | chr18 | 12599926 | 12737050 | *Ttc39c* | -3,707968306 | 8,834381996 | 2,88583E-06 |
| chr18_31804057_31908987 | chr18 | 31804057 | 31908987 | *Wdr33* | -3,209811503 | 8,193309638 | 0,00904414 |
| chr1_85246851_85255465 | chr1 | 85246851 | 85255465 | *Gm16028* | 9,35151981 | 5,900021751 | 0,024090475 |
| chr19_4510472_4621752 | chr19 | 4510472 | 4621752 | *Pcx* | -3,574335232 | 8,385112978 | 0,000286942 |
| chr19_47512982_47516578 | chr19 | 47512982 | 47516578 | *Gm19557* | 10,89816799 | 7,586933622 | 0,004041433 |
| chr19_6829085_6840601 | chr19 | 6829085 | 6840601 | *Rps6ka4* | -7,543097748 | 6,637187928 | 0,000601881 |
| chr2_98662237_98664083 | chr2 | 98662237 | 98664083 | *Gm10801* | 6,89666714 | 10,96506387 | 7,55159E-62 |
| chr2_98666547_98667301 | chr2 | 98666547 | 98667301 | *Gm10800* | 6,944244257 | 13,24452273 | 2,1161E-218 |
| chr3_18054325_18057509 | chr3 | 18054325 | 18057509 | *Bhlhe22* | -10,56310071 | 5,459748715 | 0,031763909 |
| chr3_87357881_87371074 | chr3 | 87357881 | 87371074 | *Cd5l* | 8,798658154 | 7,454408039 | 8,68128E-05 |
| chr4_108165437_108179308 | chr4 | 108165437 | 108179308 | *Echdc2* | 7,832486406 | 6,874000412 | 0,028966871 |
| chr4_136880129_136886187 | chr4 | 136880129 | 136886187 | *C1qb* | -7,110530802 | 6,774645443 | 0,001640445 |
| chr4_146449023_146470292 | chr4 | 146449023 | 146470292 | *Gm13251* | 9,925854511 | 5,521743569 | 0,032403253 |
| chr4_147136278_147457019 | chr4 | 147136278 | 147457019 | *Gm26624* | 6,228645755 | 8,205763331 | 2,52459E-11 |
| chr5_113075839_113081584 | chr5 | 113075839 | 113081584 | *Crybb3* | -6,022253083 | 6,932980048 | 0,001271325 |
| chr5_114917318_114923277 | chr5 | 114917318 | 114923277 | *Gm13822* | 6,073272609 | 6,893369327 | 0,000306033 |
| chr5_120431770_120441222 | chr5 | 120431770 | 120441222 | *Lhx5* | -6,37889882 | 5,587408615 | 0,048987405 |
| chr5_15516489_15656679 | chr5 | 15516489 | 15656679 | *Gm21847* | 3,763343537 | 7,958187692 | 0,003849756 |
| chr5_73632749_73647713 | chr5 | 73632749 | 73647713 | *Sgcb* | -5,903387381 | 6,403428526 | 0,00311244 |
| chr6_131219223_131247362 | chr6 | 131219223 | 131247362 | *Klra2* | -5,648068117 | 6,734581227 | 0,000978627 |
| chr7_127191660_127196000 | chr7 | 127191660 | 127196000 | *Cd2bp2* | -8,012067768 | 7,079640711 | 6,87923E-12 |
| chr7_28293553_28302238 | chr7 | 28293553 | 28302238 | *Dll3* | -5,830726192 | 7,357718186 | 5,36797E-06 |
| chr8_20267774_20424814 | chr8 | 20267774 | 20424814 | *2610005L07Rik* | 6,805081977 | 6,842118026 | 3,64603E-05 |
| chr8_20292477_20420781 | chr8 | 20292477 | 20420781 | *Gm26804* | 6,646008867 | 6,638582006 | 0,000361687 |
| chr9_3000282_3038313 | chr9 | 3000282 | 3038313 | *Gm26870* | 8,750785408 | 13,36697924 | 0 |
| chr9_3000922_3002330 | chr9 | 3000922 | 3002330 | *Gm10722* | 9,26468077 | 10,20332496 | 8,22289E-60 |
| chr9_3003346_3006948 | chr9 | 3003346 | 3006948 | *Gm11168* | 8,763763844 | 10,43059198 | 1,77539E-62 |
| chr9_3015654_3017210 | chr9 | 3015654 | 3017210 | *Gm10720* | 12,31296096 | 6,926367318 | 1,20983E-10 |
| chr9_3017408_3021593 | chr9 | 3017408 | 3021593 | *Gm10719* | 8,589645834 | 9,192448379 | 4,44047E-31 |
| chr9_3023547_3025218 | chr9 | 3023547 | 3025218 | *Gm10718* | 8,006741452 | 10,98695999 | 3,42465E-74 |
| chr9_3025417_3033289 | chr9 | 3025417 | 3033289 | *Gm10717* | 9,353116186 | 10,91265059 | 1,07585E-88 |
| chr9_3034599_3035805 | chr9 | 3034599 | 3035805 | *Gm17535* | 13,92422298 | 8,413677184 | 3,86757E-29 |

| | | | | | | | |
|---|---|---|---|---|---|---|---|
| chr9_3036877_3038316 | chr9 | 3036877 | 3038316 | *Gm10715* | 12,48409232 | 6,896222485 | 9,22504E-11 |
| chrX_103083003_103083250 | chrX | 103083003 | 103083250 | *Gm14838* | -11,36443398 | 5,700875423 | 0,025784341 |
