# Supplementary material for: Identification and Characterization of Extracellular Vesicles and Its DNA Cargo Secreted During Murine Embryo Development
Source: Genes (Basel). 2020 Feb 17;11(2):203. doi: 10.3390/genes11020203 (PMC7074575; doi:10.3390/genes11020203)
Supplement: Supplementary file 1 [file genes-11-00203-s001.zip › Table S7.pdf]

Table S7. Sequencing analysis data for the enriched genes in the comparison ABs vs blank control for gene sequences uniquely binding the *Mus musculus* reference genome.

id	chr	start	end	gene	logFC	logCPM	FWER
chr10_118860826_118863542	chr10	118860826	118863542	*4932442E05Rik*	-11,44497403	5,490516418	5,30905E-05
chr10_60302748_60696490	chr10	60302748	60696490	*Cdh23*	1,649099291	11,14954224	0,001003649
chr11_117966079_117970047	chr11	117966079	117970047	*Socs3*	-10,60460116	5,513478721	0,011288099
chr11_3125904_3131004	chr11	3125904	3131004	*Pisd-ps1*	11,80927499	7,546045555	1,50798E-13
chr11_3131850_3193463	chr11	3131850	3193463	*Sfi1*	4,587147054	10,26359019	3,10764E-21
chr11_3142048_3143363	chr11	3142048	3143363	*Gm11399*	9,961999811	5,885694338	0,007977605
chr12_85110833_85134091	chr12	85110833	85134091	*Dlst*	-4,7862089	7,350444007	0,001652127
chr13_119487941_119610458	chr13	119487941	119610458	*Gm7120*	7,615347032	8,016567803	2,89096E-13
chr13_119606650_119610458	chr13	119606650	119610458	*Gm21967*	10,6929194	6,493150042	0,000116243
chr13_40715675_40733823	chr13	40715675	40733823	*Tfap2a*	-8,343717347	6,112572126	0,002283518
chr13_55825051_55836192	chr13	55825051	55836192	*Pitx1*	-8,588552816	5,182515654	0,025569443
chr14_103080239_103099566	chr14	103080239	103099566	*Fbxl3*	-8,448893965	5,336815217	0,008721141
chr14_19415857_19418930	chr14	19415857	19418930	*Gm21738*	6,435395652	11,27633001	9,43189E-63
chr15_78934933_78943634	chr15	78934933	78943634	*Nol12*	-9,232547269	5,31789469	0,004980914
chr16_17646470_17650738	chr16	17646470	17650738	*Ccdc74a*	-10,95546274	5,291543693	0,019483114
chr16_3858321_3884561	chr16	3858321	3884561	*Zfp597*	-3,224581974	8,274119125	0,02641821
chr16_3862384_3884505	chr16	3862384	3884505	*Gm26675*	-3,211423001	8,233242076	0,042689523
chr16_3872375_3904770	chr16	3872375	3904770	*Naa60*	-3,664784093	8,18896789	0,002872563
chr17_3076578_3084183	chr17	3076578	3084183	*Pisd-ps2*	8,428946455	7,019574733	1,50917E-07
chr18_12599926_12737050	chr18	12599926	12737050	*Ttc39c*	-3,997609778	8,834381996	1,66364E-06
chr19_4510472_4621752	chr19	4510472	4621752	*Pcx*	-3,068176955	8,385112978	0,040579582
chr2_129493570_129515807	chr2	129493570	129515807	*Gm14040*	6,287116982	6,60815214	0,000672688
chr2_98662237_98664083	chr2	98662237	98664083	*Gm10801*	5,573431001	10,96506387	1,7983E-44
chr2_98666547_98667301	chr2	98666547	98667301	*Gm10800*	5,398501235	13,24452273	1,6303E-147
chr3_18054325_18057509	chr3	18054325	18057509	*Bhlhe22*	-11,18760684	5,459748715	0,005758204
chr3_87357881_87371074	chr3	87357881	87371074	*Cd5l*	9,477049379	7,454408039	1,75626E-06
chr3_89767511_89773240	chr3	89767511	89773240	*4632404H12Rik*	-10,90830077	4,763813185	0,009061917
chr4_101647718_101659364	chr4	101647718	101659364	*Leprot*	8,029721427	5,44621344	0,044954295
chr4_136880129_136886187	chr4	136880129	136886187	*C1qb*	-7,609111026	6,774645443	0,004295518
chr4_146449023_146470292	chr4	146449023	146470292	*Gm13251*	10,27716383	5,521743569	0,016575473
chr4_147136278_147457019	chr4	147136278	147457019	*Gm26624*	6,592050641	8,205763331	2,87695E-12
chr5_113075839_113081584	chr5	113075839	113081584	*Crybb3*	-6,127593361	6,932980048	0,006103303
chr5_114917318_114923277	chr5	114917318	114923277	*Gm13822*	5,87168909	6,893369327	0,000755277
chr5_120431770_120441222	chr5	120431770	120441222	*Lhx5*	-8,788398857	5,587408615	0,002781808
chr5_120861421_120873512	chr5	120861421	120873512	*Oas1h*	9,819822415	6,052533243	0,006867565
chr5_15516489_15656679	chr5	15516489	15656679	*Gm21847*	4,239263018	7,958187692	0,000280404
chr5_24751003_24758008	chr5	24751003	24758008	*Crygn*	-7,422898455	5,603219896	0,015919862
chr6_119479668_119483886	chr6	119479668	119483886	*Fbxl14*	-11,28156843	4,170098997	0,035451902
chr6_131219223_131247362	chr6	131219223	131247362	*Klra2*	-8,807357173	6,734581227	1,90282E-08
chr6_132855438_132856355	chr6	132855438	132856355	*Tas2r116*	-11,41464129	4,725382268	0,005321471
chr7_10158652_10176286	chr7	10158652	10176286	*Vmn2r52*	-11,20601073	5,485537521	0,000298253
chr7_127191660_127196000	chr7	127191660	127196000	*Cd2bp2*	-10,17244457	7,079640711	5,02468E-13
chr7_26061495_26096196	chr7	26061495	26096196	*Cyp2b13*	4,973740516	6,992510956	0,007538791
chr7_28293553_28302238	chr7	28293553	28302238	*Dll3*	-5,932909923	7,357718186	5,25182E-05
chr7_80429210_80430740	chr7	80429210	80430740	*Gm15544*	8,468435843	7,129400261	0,00228763
chr8_20267774_20424814	chr8	20267774	20424814	*2610005L07Rik*	6,85111491	6,842118026	5,45057E-05
chr8_20292477_20420781	chr8	20292477	20420781	*Gm26804*	6,849295066	6,638582006	0,000298566
chr8_22974844_23150497	chr8	22974844	23150497	*Ank1*	3,13576689	8,412277131	0,018561041

chr9_3000282_3038313	chr9	3000282	3038313	*Gm26870*	6,67962923	13,36697924	4,1606E-219
chr9_3000922_3002330	chr9	3000922	3002330	*Gm10722*	7,132626176	10,20332496	1,7039E-40
chr9_3003346_3006948	chr9	3003346	3006948	*Gm11168*	6,689037227	10,43059198	1,8761E-41
chr9_3015654_3017210	chr9	3015654	3017210	*Gm10720*	10,34489138	6,926367318	7,78641E-07
chr9_3017408_3021593	chr9	3017408	3021593	*Gm10719*	6,314750201	9,192448379	1,21113E-18
chr9_3023547_3025218	chr9	3023547	3025218	*Gm10718*	5,899088857	10,98695999	5,67399E-46
chr9_3025417_3033289	chr9	3025417	3033289	*Gm10717*	7,352998505	10,91265059	1,9642E-62
chr9_3034599_3035805	chr9	3034599	3035805	*Gm17535*	11,57642475	8,413677184	3,9047E-20
chr9_3036877_3038316	chr9	3036877	3038316	*Gm10715*	10,07172229	6,896222485	3,28643E-06
chrX_103083003_103083250	chrX	103083003	103083250	*Gm14838*	-11,61920642	5,700875423	0,00709982
