# Supplementary material for: Identification and Characterization of Extracellular Vesicles and Its DNA Cargo Secreted During Murine Embryo Development
Source: Genes (Basel). 2020 Feb 17;11(2):203. doi: 10.3390/genes11020203 (PMC7074575; doi:10.3390/genes11020203)
Supplement: Supplementary file 1 [file genes-11-00203-s001.zip › Table S8.pdf]

Table S8. Sequencing analysis data for the enriched genes in the comparison naEVs vs blank control for gene sequences uniquely binding the *Mus musculus* reference genome.

id	chr	start	end	gene	logFC	logCPM	FWER
chr11_116280939_116283938	chr11	116280939	116283938	*Galr2*	-5.951659478	6.762493549	0.003265658
chr11_3125904_3131004	chr11	3125904	3131004	*Pisd-ps1*	11.51870399	7.546045555	6.04819E-13
chr11_3131850_3193463	chr11	3131850	3193463	*Sfi1*	4.791174808	10.26359019	1.59001E-23
chr11_3142048_3143363	chr11	3142048	3143363	*Gm11399*	10.12007364	5.885694338	0.004013355
chr1_191351981_191397041	chr1	191351981	191397041	*Ppp2r5a*	-5.173465671	7.160724603	0.00510022
chr11_97362551_97398542	chr11	97362551	97398542	*Socs7*	-3.636715773	7.700942682	0.007489596
chr12_112588784_112615556	chr12	112588784	112615556	*Inf2*	-5.03402328	6.855061312	0.03737192
chr12_85110833_85134091	chr12	85110833	85134091	*Dlst*	-4.352699034	7.350444007	0.013846499
chr13_119487941_119610458	chr13	119487941	119610458	*Gm7120*	7.895964085	8.016567803	5.79003E-14
chr13_119606650_119610458	chr13	119606650	119610458	*Gm21967*	12.54560826	6.493150042	1.42339E-07
chr14_19415857_19418930	chr14	19415857	19418930	*Gm21738*	7.141398219	11.27633001	1.29072E-74
chr1_51987148_52107188	chr1	51987148	52107188	*Stat4*	-3.950010419	8.015519032	0.000636886
chr15_78934933_78943634	chr15	78934933	78943634	*Nol12*	-6.782239902	5.31789469	0.048356851
chr16_3858321_3884561	chr16	3858321	3884561	*Zfp597*	-4.949762896	8.274119125	6.9456E-10
chr16_3862384_3884505	chr16	3862384	3884505	*Gm26675*	-5.259544986	8.233242076	1.8721E-10
chr16_3872375_3904770	chr16	3872375	3904770	*Naa60*	-4.89367574	8.18896789	3.4796E-09
chr1_66924295_66945404	chr1	66924295	66945404	*Myl1*	-5.143499332	6.768925192	0.01192866
chr17_3076578_3084183	chr17	3076578	3084183	*Pisd-ps2*	7.979550831	7.019574733	6.26906E-07
chr18_12599926_12737050	chr18	12599926	12737050	*Ttc39c*	-3.620377591	8.834381996	9.73476E-06
chr18_31804057_31908987	chr18	31804057	31908987	*Wdr33*	-2.95824482	8.193309638	0.038910843
chr19_42755105_42779978	chr19	42755105	42779978	*Hps1*	-4.312112739	7.005513375	0.017013678
chr19_44548572_44555440	chr19	44548572	44555440	*Ndufb8*	-7.307537509	5.400361398	0.008757844
chr19_4510472_4621752	chr19	4510472	4621752	*Pcx*	-3.532947685	8.385112978	0.000318327
chr19_47512982_47516578	chr19	47512982	47516578	*Gm19557*	10.94331846	7.586933622	0.011684977
chr19_6829085_6840601	chr19	6829085	6840601	*Rps6ka4*	-7.017000754	6.637187928	0.020318911
chr2_98662237_98664083	chr2	98662237	98664083	*Gm10801*	6.228353651	10.96506387	2.5356E-53
chr2_98666547_98667301	chr2	98666547	98667301	*Gm10800*	5.989227147	13.24452273	2.3875E-175
chr3_87357881_87371074	chr3	87357881	87371074	*Cd5l*	9.500628601	7.454408039	2.09256E-06
chr4_107126613_107136252	chr4	107126613	107136252	*Gm12802*	-7.746999557	5.614033243	0.000243199
chr4_108165437_108179308	chr4	108165437	108179308	*Echdc2*	7.899424042	6.874000412	0.027120545
chr4_133964738_133968650	chr4	133964738	133968650	*Hmgn2*	-10.32138708	4.889345786	0.031677526
chr4_136880129_136886187	chr4	136880129	136886187	*C1qb*	-6.517603507	6.774645443	0.023710039
chr4_146449023_146470292	chr4	146449023	146470292	*Gm13251*	10.34852498	5.521743569	0.012245168
chr4_147136278_147457019	chr4	147136278	147457019	*Gm26624*	6.120195604	8.205763331	5.62323E-11
chr5_113075839_113081584	chr5	113075839	113081584	*Crybb3*	-6.238322052	6.932980048	0.000541336
chr5_114917318_114923277	chr5	114917318	114923277	*Gm13822*	6.124761839	6.893369327	0.000211117
chr5_15516489_15656679	chr5	15516489	15656679	*Gm21847*	3.955896544	7.958187692	0.001187405
chr5_73632749_73647713	chr5	73632749	73647713	*Sgcb*	-5.918119779	6.403428526	0.00353774
chr6_131219223_131247362	chr6	131219223	131247362	*Klra2*	-5.597463376	6.734581227	0.001854914
chr6_132855438_132856355	chr6	132855438	132856355	*Tas2r116*	-10.91701075	4.725382268	0.010994983
chr6_143167230_143208542	chr6	143167230	143208542	*Etnk1*	-3.876651486	7.442291552	0.030187678
chr7_127191660_127196000	chr7	127191660	127196000	*Cd2bp2*	-7.26994817	7.079640711	1.94102E-10
chr7_28293553_28302238	chr7	28293553	28302238	*Dll3*	-6.487078517	7.357718186	6.04774E-08
chr7_80429210_80430740	chr7	80429210	80430740	*Gm15544*	8.266849146	7.129400261	0.00239783
chr8_20267774_20424814	chr8	20267774	20424814	*2610005L07Rik*	6.482496587	6.842118026	0.000157169
chr8_20292477_20420781	chr8	20292477	20420781	*Gm26804*	6.417031477	6.638582006	0.000935245
chr9_3000282_3038313	chr9	3000282	3038313	*Gm26870*	7.340336564	13.36697924	5.7842E-256
chr9_3000922_3002330	chr9	3000922	3002330	*Gm10722*	7.851197209	10.20332496	1.78081E-47
chr9_3003346_3006948	chr9	3003346	3006948	*Gm11168*	7.320062285	10.43059198	1.82182E-48
chr9_3015654_3017210	chr9	3015654	3017210	*Gm10720*	10.91757068	6.926367318	4.95127E-08
chr9_3017408_3021593	chr9	3017408	3021593	*Gm10719*	7.01669075	9.192448379	6.71884E-23
chr9_3023547_3025218	chr9	3023547	3025218	*Gm10718*	6.528125684	10.98695999	4.87891E-55
chr9_3025417_3033289	chr9	3025417	3033289	*Gm10717*	8.01507627	10.91265059	5.00392E-72

chr9_3034599_3035805	chr9	3034599	3035805	*Gm17535*	12.27860312	8.413677184	5.09085E-23
chr9_3036877_3038316	chr9	3036877	3038316	*Gm10715*	10.71903952	6.896222485	1.53442E-07
chrX_103083003_103083250	chrX	103083003	103083250	*Gm14838*	-11.08848988	5.700875423	0.017657604
