# Supplementary material for: Identification and Characterization of Extracellular Vesicles and Its DNA Cargo Secreted During Murine Embryo Development
Source: Genes (Basel). 2020 Feb 17;11(2):203. doi: 10.3390/genes11020203 (PMC7074575; doi:10.3390/genes11020203)
Supplement: Supplementary file 1 [file genes-11-00203-s001.zip › Table S9.pdf]

Table S9. Sequencing analysis data for the enriched genes in the comparison SN vs blank control for gene sequences uniquely binding the *Mus musculus* reference genome.

id	chr	start	end	gene	logFC	logCPM	FWER
chr10_122678762_122945795	chr10	122678762	122945795	*Ppm1h*	3,021867878	9,016642224	0,002221176
chr10_127525473_127534559	chr10	127525473	127534559	*Nxph4*	8,714818747	6,251114639	0,007421448
chr10_42740539_42758660	chr10	42740539	42758660	*Gm15199*	5,947590086	7,225915611	0,000127542
chr10_60302748_60696490	chr10	60302748	60696490	*Cdh23*	1,948144631	11,14954224	3,97675E-06
chr10_81292963_81319973	chr10	81292963	81319973	*Pip5k1c*	4,165660322	7,876645963	0,036704001
chr10_81606270_81620703	chr10	81606270	81620703	*BC025920*	6,770304422	6,992024552	0,001853612
chr10_81606368_81609791	chr10	81606368	81609791	*Gm26896*	8,515520383	6,920303216	0,004733443
chr10_93939165_94035817	chr10	93939165	94035817	*Vezt*	4,455336642	8,093543263	0,001398659
chr11_101367561_101377903	chr11	101367561	101377903	*G6pc*	11,14270919	5,589509746	0,005467388
chr11_102189620_102194081	chr11	102189620	102194081	*G6pc3*	-11,03901433	5,591848559	0,013153547
chr11_11557421_11579460	chr11	11557421	11579460	*Gm11998*	7,91787874	7,207639709	3,94419E-06
chr11_119228572_119265236	chr11	119228572	119265236	*Ccdc40*	5,502141535	9,373923275	3,0211E-11
chr11_120007313_120047167	chr11	120007313	120047167	*Aatk*	4,116809758	8,43121019	0,000745711
chr11_120064430_120086827	chr11	120064430	120086827	*Azi1*	6,527221649	7,69616468	2,97035E-05
chr11_20685084_20741589	chr11	20685084	20741589	*Aftph*	4,036386185	9,021198482	1,45357E-05
chr11_3125904_3131004	chr11	3125904	3131004	*Pisd-ps1*	11,03437842	7,546045555	1,51647E-11
chr11_3131850_3193463	chr11	3131850	3193463	*Sfi1*	5,461594399	10,26359019	6,45896E-29
chr11_3142048_3143363	chr11	3142048	3143363	*Gm11399*	10,01045642	5,885694338	0,00776851
chr11_50174444_50200115	chr11	50174444	50200115	*3010026O09Rik*	8,4689081	6,155254074	0,000913418
chr11_69183932_69197843	chr11	69183932	69197843	*Alox8*	7,897638413	6,677223736	0,000610947
chr11_80046493_80080991	chr11	80046493	80080991	*Crlf3*	7,245023449	6,888126572	9,13013E-05
chr1_180568955_180601253	chr1	180568955	180601253	*Parp1*	8,081431993	8,142560563	5,82567E-12
chr1_180942344_180975112	chr1	180942344	180975112	*Tmem63a*	4,635829021	7,817913194	0,009310733
chr11_82942341_82960681	chr11	82942341	82960681	*1700003F17Rik*	6,98789459	5,885284081	0,02908363
chr1_182954788_182976044	chr1	182954788	182976044	*Tlr5*	7,089506571	8,029790085	5,17347E-08
chr11_8826708_8973266	chr11	8826708	8973266	*Pkd1l1*	4,99258433	9,132365413	3,43106E-09
chr12_73584797_73778185	chr12	73584797	73778185	*Prkch*	3,231444359	8,607248121	0,016002699
chr13_105149352_105271039	chr13	105149352	105271039	*Rnf180*	4,381884897	8,466768284	3,34239E-05
chr13_119487941_119610458	chr13	119487941	119610458	*Gm7120*	7,780541523	8,016567803	1,39897E-13
chr13_53109312_53286124	chr13	53109312	53286124	*Ror2*	3,853383391	9,469311176	1,7036E-08
chr13_64058274_64089201	chr13	64058274	64089201	*Hsd17b3*	5,728701157	7,280433308	0,000766119
chr13_93674433_93752831	chr13	93674433	93752831	*Dmgdh*	4,653358324	8,28224771	0,000726287
chr14_19415857_19418930	chr14	19415857	19418930	*Gm21738*	7,546367454	11,27633001	2,01944E-80
chr14_32408203_32465909	chr14	32408203	32465909	*Chat*	4,152679748	8,13374732	0,033443779
chr14_44858671_44974944	chr14	44858671	44974944	*Gm16976*	4,269409076	8,246992418	0,000226064
chr14_64652531_64806296	chr14	64652531	64806296	*Kif13b*	3,229211226	8,736018234	0,026754231
chr14_66140960_66152948	chr14	66140960	66152948	*Chrna2*	5,509175364	6,569853936	0,025014668
chr15_4898737_4962203	chr15	4898737	4962203	*Mroh2b*	4,599687368	7,869412262	0,027491853
chr15_74606029_74636298	chr15	74606029	74636298	*Mroh4*	5,401858871	8,325299657	3,0263E-08
chr15_83357526_83367282	chr15	83357526	83367282	*1700001L05Rik*	10,685279	5,616886888	0,016507372
chr16_33829665_33949153	chr16	33829665	33949153	*Itgb5*	3,40355725	8,709172393	0,023413846
chr17_3076578_3084183	chr17	3076578	3084183	*Pisd-ps2*	7,210290328	7,019574733	2,48484E-05
chr17_37001163_37010635	chr17	37001163	37010635	*Zfp57*	10,54784705	5,107017647	0,047349435
chr17_53716509_53733843	chr17	53716509	53733843	*4932415M13Rik*	11,71032155	6,209777336	5,94833E-05
chr18_10781723_10785883	chr18	10781723	10785883	*Gm21747*	12,34681532	6,487111346	8,47286E-06
chr18_12599926_12737050	chr18	12599926	12737050	*Ttc39c*	-3,244562771	8,834381996	0,004261075
chr18_36281097_36301521	chr18	36281097	36301521	*Pura*	6,962909968	7,754360341	1,13489E-05
chr1_85246851_85255465	chr1	85246851	85255465	*Gm16028*	11,09060846	5,900021751	0,000890525

chr19_43956307_43986556	chr19	43956307	43986556	*Cpn1*	5,573481293	6,951906771	0,008338811
chr19_47501033_47537507	chr19	47501033	47537507	*Obfc1*	4,486536362	7,848776646	0,00181191
chr19_47512982_47516578	chr19	47512982	47516578	*Gm19557*	12,34043535	7,586933622	2,29441E-07
chr19_5298331_5308738	chr19	5298331	5308738	*Gal3st3*	5,318040572	6,575480927	0,044557652
chr19_6062821_6068373	chr19	6062821	6068373	*Tm7sf2*	6,181181526	6,575242584	0,028223583
chr19_7092014_7105729	chr19	7092014	7105729	*Flrt1*	10,03320356	7,092658857	8,18872E-09
chr19_8591254_8611834	chr19	8591254	8611834	*Slc22a8*	6,154424168	6,411529411	0,020908666
chr2_172393794_172433757	chr2	172393794	172433757	*Cass4*	3,789808102	8,481844903	0,000581211
chr2_23068168_23114510	chr2	23068168	23114510	*Acbd5*	7,091393483	7,014908498	9,15384E-05
chr2_28315016_28324050	chr2	28315016	28324050	*Gm13373*	11,70127308	6,434235174	3,98433E-05
chr2_84330626_84425411	chr2	84330626	84425411	*Calcrl*	-5,171987713	6,819864457	0,03594058
chr2_98662237_98664083	chr2	98662237	98664083	*Gm10801*	6,73632588	10,96506387	3,25817E-60
chr2_98666547_98667301	chr2	98666547	98667301	*Gm10800*	6,46390988	13,24452273	5,6775E-199
chr3_10417819_10440124	chr3	10417819	10440124	*Snx16*	4,976827528	7,157372382	0,044991006
chr3_18054325_18057509	chr3	18054325	18057509	*Bhlhe22*	-11,15937646	5,459748715	0,039286607
chr3_20248264_20275733	chr3	20248264	20275733	*Cpb1*	5,309179248	8,537871086	9,30293E-08
chr3_30995747_31052741	chr3	30995747	31052741	*Prkci*	4,796718472	7,56758201	0,00064231
chr3_85574119_85655622	chr3	85574119	85655622	*Pet112*	5,14664974	7,678997721	0,000470219
chr3_87357881_87371074	chr3	87357881	87371074	*Cd5l*	12,6803478	7,454408039	4,63159E-13
chr4_107134155_107179116	chr4	107134155	107179116	*Tceanc2*	5,114040641	9,025613449	4,07866E-09
chr4_108165437_108179308	chr4	108165437	108179308	*Echdc2*	12,83475508	6,874000412	9,45949E-09
chr4_125490700_125714173	chr4	125490700	125714173	*Grik3*	3,325940061	9,432653014	3,36928E-05
chr4_134065912_134092504	chr4	134065912	134092504	*Aim1l*	4,932907253	7,310376945	0,049070619
chr4_136880129_136886187	chr4	136880129	136886187	*C1qb*	-9,62372559	6,774645443	1,97935E-06
chr4_139653538_139670280	chr4	139653538	139670280	*Tas1r2*	5,712264735	7,380261261	0,002494452
chr4_147136278_147457019	chr4	147136278	147457019	*Gm26624*	5,596658334	8,205763331	4,51251E-09
chr4_148558429_148582401	chr4	148558429	148582401	*Exosc10*	5,651506495	6,810816645	0,00901861
chr4_155260129_155361361	chr4	155260129	155361361	*Prkcz*	5,36435476	8,936716226	3,35024E-10
chr4_72117142_72200919	chr4	72117142	72200919	*Tle1*	4,066391059	8,375952259	0,004565679
chr5_114917318_114923277	chr5	114917318	114923277	*Gm13822*	5,865934452	6,893369327	0,000784734
chr5_120861421_120873512	chr5	120861421	120873512	*Oas1h*	11,71091706	6,052533243	3,01118E-05
chr5_125277087_125341094	chr5	125277087	125341094	*Scarb1*	4,759738825	7,372061119	0,023119428
chr5_25759997_25850342	chr5	25759997	25850342	*Actr3b*	5,704221812	8,292123251	1,22554E-07
chr5_35041553_35048436	chr5	35041553	35048436	*Hgfac*	6,700607755	6,782447523	0,017940977
chr6_113471427_113483140	chr6	113471427	113483140	*Il17rc*	6,997670729	6,668080174	0,001306456
chr6_116208033_116262668	chr6	116208033	116262668	*Fam21*	5,875100794	7,569363118	4,05305E-05
chr6_121838541_121889057	chr6	121838541	121889057	*Mug1*	5,03100315	7,30234535	0,001791221
chr6_37327255_37442146	chr6	37327255	37442146	*Creb3l2*	3,377520967	9,073866505	0,016515874
chr6_48130918_48395408	chr6	48130918	48395408	*Gm16630*	3,658281726	8,110438719	0,016857867
chr6_49036518_49058182	chr6	49036518	49058182	*Gpnmb*	5,492948551	7,934191386	2,87677E-06
chr6_91464276_91473423	chr6	91464276	91473423	*Chchd4*	6,545174437	7,456994739	0,00176444
chr7_122670492_122769393	chr7	122670492	122769393	*Cacng3*	3,769514826	8,200921226	0,031470439
chr7_127191660_127196000	chr7	127191660	127196000	*Cd2bp2*	-4,888420061	7,079640711	0,00923379
chr7_25619414_25625551	chr7	25619414	25625551	*Atp5sl*	12,44607539	5,642912591	0,003145368
chr7_45976380_46030302	chr7	45976380	46030302	*Abcc6*	5,095581346	7,25715469	0,027687467
chr7_66390892_66427517	chr7	66390892	66427517	*Aldh1a3*	4,866812079	8,330230401	5,01933E-05
chr7_80429210_80430740	chr7	80429210	80430740	*Gm15544*	12,72185126	7,129400261	2,92433E-09
chr7_83584927_83625614	chr7	83584927	83625614	*Tmc3*	4,86193816	8,932640185	1,42351E-05
chr7_83597048_83631842	chr7	83597048	83631842	*Gm16638*	4,967137604	8,569070686	0,000120927
chr8_109859984_109868455	chr8	109859984	109868455	*Gm17344*	8,884146659	5,786345715	0,032711463
chr8_111345135_111393824	chr8	111345135	111393824	*Fa2h*	6,046422878	7,631259735	6,01945E-05
chr8_122578238_122611487	chr8	122578238	122611487	*Galns*	5,717186459	7,633220302	0,000107553
chr8_13465374_13494535	chr8	13465374	13494535	*Gas6*	8,946049906	7,996229119	1,42969E-13

chr8_20267774_20424814	chr8	20267774	20424814	*2610005L07Rik*	5,977425398	6,842118026	0,001313349
chr8_20292477_20420781	chr8	20292477	20420781	*Gm26804*	5,437449078	6,638582006	0,039547756
chr8_70302518_70312993	chr8	70302518	70312993	*Cope*	8,759639297	6,658048523	0,000332194
chr8_71396855_71404772	chr8	71396855	71404772	*Babam1*	4,924988231	7,272610284	0,004334006
chr8_74590987_74833947	chr8	74590987	74833947	*Gm11033*	2,914082792	9,263333906	0,010363723
chr8_84990595_85003364	chr8	84990595	85003364	*Hook2*	5,88758878	6,926564662	0,010979108
chr9_110865711_110880113	chr9	110865711	110880113	*Tmie*	7,434424585	8,078489759	1,76821E-11
chr9_111439081_111489608	chr9	111439081	111489608	*Dclk3*	4,418472695	8,131332646	0,000358773
chr9_20468549_20492746	chr9	20468549	20492746	*Zfp426*	6,8709592	7,334615754	1,0353E-05
chr9_20770050_20815067	chr9	20770050	20815067	*Col5a3*	5,439785043	8,146594632	5,51004E-06
chr9_3000282_3038313	chr9	3000282	3038313	*Gm26870*	7,652692948	13,36697924	4,4341E-270
chr9_3000922_3002330	chr9	3000922	3002330	*Gm10722*	8,158350957	10,20332496	5,01936E-50
chr9_3003346_3006948	chr9	3003346	3006948	*Gm11168*	7,577367218	10,43059198	1,23587E-50
chr9_3015654_3017210	chr9	3015654	3017210	*Gm10720*	11,16908851	6,926367318	2,13503E-08
chr9_3017408_3021593	chr9	3017408	3021593	*Gm10719*	7,330335759	9,192448379	2,07774E-24
chr9_3023547_3025218	chr9	3023547	3025218	*Gm10718*	6,774808275	10,98695999	2,76481E-58
chr9_3025417_3033289	chr9	3025417	3033289	*Gm10717*	8,332025627	10,91265059	1,25301E-75
chr9_3034599_3035805	chr9	3034599	3035805	*Gm17535*	12,48411074	8,413677184	1,48102E-23
chr9_3036877_3038316	chr9	3036877	3038316	*Gm10715*	11,03249816	6,896222485	5,43135E-08
chr9_56135885_56161070	chr9	56135885	56161070	*Tspan3*	6,461323641	7,744852074	1,99372E-08
chrX_133891068_133898429	chrX	133891068	133898429	*Tspan6*	-7,474031354	6,553125517	0,019045655
chrX_38013173_38018815	chrX	38013173	38018815	*4930525M21Rik*	11,00951128	5,77509237	0,011027024
chrX_38013908_38018815	chrX	38013908	38018815	*4930525M21RIK*	11,01227313	5,770080356	0,011225712
