# Supplementary figures and images for: Identification and Characterization of Extracellular Vesicles and Its DNA Cargo Secreted During Murine Embryo Development
Source: Genes (Basel). 2020 Feb 17;11(2):203. doi: 10.3390/genes11020203 (PMC7074575; doi:10.3390/genes11020203)

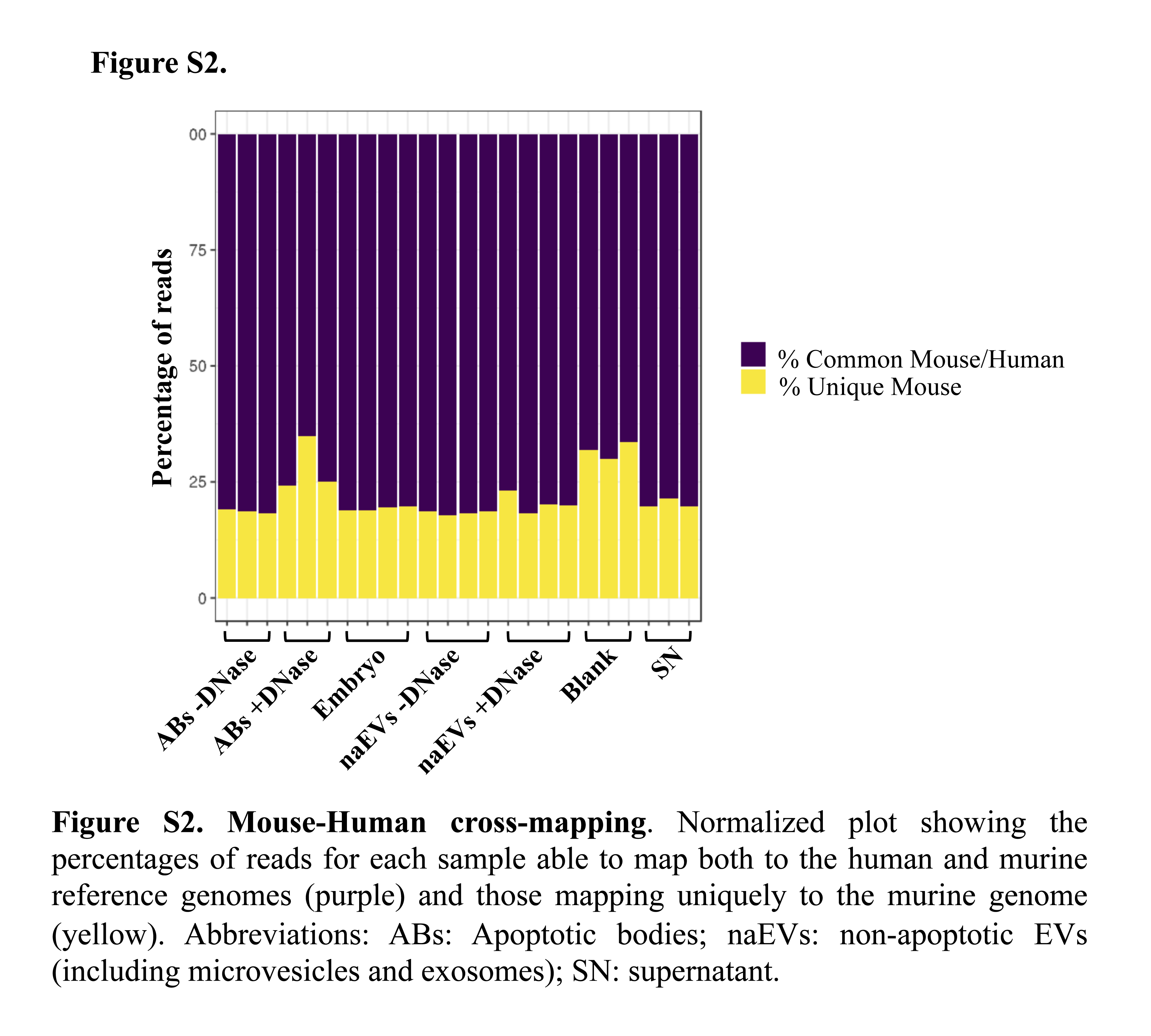

Supplement: Supplementary file 1 [file genes-11-00203-s001.zip › Figure S2.tif]
